# Genomic and Genetic Insights Into a Cosmopolitan Fungus, *Paecilomyces variotii* (Eurotiales)

**DOI:** 10.3389/fmicb.2018.03058

**Published:** 2018-12-13

**Authors:** Andrew S. Urquhart, Stephen J. Mondo, Miia R. Mäkelä, James K. Hane, Ad Wiebenga, Guifen He, Sirma Mihaltcheva, Jasmyn Pangilinan, Anna Lipzen, Kerrie Barry, Ronald P. de Vries, Igor V. Grigoriev, Alexander Idnurm

**Affiliations:** ^1^School of BioSciences, University of Melbourne, Melbourne, VIC, Australia; ^2^U.S. Department of Energy Joint Genome Institute, Walnut Creek, CA, United States; ^3^Department of Microbiology, Faculty of Agriculture and Forestry, Viikki Biocenter 1, University of Helsinki, Helsinki, Finland; ^4^CCDM Bioinformatics, Centre for Crop and Disease Management, Curtin University, Bentley, WA, Australia; ^5^Curtin Institute for Computation, Curtin University, Bentley, WA, Australia; ^6^Fungal Physiology, Westerdijk Fungal Biodiversity Institute and Fungal Molecular Physiology, Utrecht University, Utrecht, Netherlands

**Keywords:** *Agrobacterium tumefaciens*-mediated transformation, *Byssochlamys spectabilis*, DUF1212, Eurotiales, genome defense, *leuA*, mitochondrial membrane carrier

## Abstract

Species in the genus *Paecilomyces*, a member of the fungal order Eurotiales, are ubiquitous in nature and impact a variety of human endeavors. Here, the biology of one common species, *Paecilomyces variotii*, was explored using genomics and functional genetics. Sequencing the genome of two isolates revealed key genome and gene features in this species. A striking feature of the genome was the two-part nature, featuring large stretches of DNA with normal GC content separated by AT-rich regions, a hallmark of many plant-pathogenic fungal genomes. These AT-rich regions appeared to have been mutated by repeat-induced point (RIP) mutations. We developed methods for genetic transformation of *P. variotii*, including forward and reverse genetics as well as crossing techniques. Using transformation and crossing, RIP activity was identified, demonstrating for the first time that RIP is an active process within the order Eurotiales. A consequence of RIP is likely reflected by a reduction in numbers of genes within gene families, such as in cell wall degradation, and reflected by growth limitations on *P. variotii* on diverse carbon sources. Furthermore, using these transformation tools we characterized a conserved protein containing a domain of unknown function (DUF1212) and discovered it is involved in pigmentation.

## Introduction

Species in the order Eurotiales are amongst some of the best characterized fungi. They include the source of life-saving penicillin *Penicillium rubens*, the model filamentous fungus *Aspergillus nidulans*, the industrial species and source of citric acid *Aspergillus niger*, and the human pathogen *Aspergillus fumigatus* (Galagan et al., 2005; Max et al., 2010; de Vries et al., 2017). While a handful of these species have been extensively studied most have not received a high level of investigation, yet might provide similar benefits or risks to people.

*Paecilomyces variotii* is a ubiquitous thermo-tolerant species that is encountered in food products, soil, indoor environments and clinical samples (Houbraken et al., 2010). Its thermo-tolerance and ability to grow at low oxygen levels allows it to survive heat treatment and it has been widely isolated as a contaminant of products such as heat-treated fruit juices (Houbraken et al., 2006). Furthermore, it is emerging as an opportunistic human pathogen (Steiner et al., 2013), with cases of *P. variotii* and the closely related species *Paecilomyces formosus* infection in immuno-compromised individuals (Torres et al., 2014; Polat et al., 2015; Feldman et al., 2016; Kuboi et al., 2016; Swami et al., 2016; Bellanger et al., 2017; Uzunoglu and Sahin, 2017) and plant disease (Heidarian et al., 2018). While this organism can be detrimental to human health, it also lends itself to diverse industrial applications. *P. variotii* has been explored as a source of industrial tannase, as its tannase has beneficial characteristics including a high optimum temperature (Battestin and Macedo, 2007a,b). Among its other enzymes with favorable properties for industry are a thermostable glucoamylase (Michelin et al., 2008), a glucose-tolerant β-glucosidase (Job et al., 2010) and an alcohol oxidase that displays stability at high temperature (50°C) and over a wide pH range (from 5 to 10) (Kondo et al., 2008).

Despite the relevance of *Paecilomyces* species to human activities across the world, no well-annotated genome sequence is currently available for any members in the *Paecilomyces* genus except for draft genomes of *P. formosus* (Oka et al., 2014) and *P. niveus* (Biango-Daniels et al., 2018). Furthermore, methods for genetic manipulation or classical genetics have not been described for *Paecilomyces*, further limiting our ability to understand gene functions in the genus.

Here, we sequenced and annotated the genome of *P. variotii* [*Byssochlamys spectabilis*] CBS 101075, which is the type strain of the teleomorphic state (Houbraken et al., 2008), and strain CBS 144490 that was isolated in this study. The genomes have a bi-modal pattern of overall DNA G:C content with alternating stretches of G:C-equilibrated or A:T rich DNA, reminiscent of those found in the genomes of many plant pathogens as a consequence of repeat induced point mutation (RIP) (Testa et al., 2016). RIP is a fungal process in which repetitive sequences are recognized during the sexual cycle and targeted for mutation (Hane et al., 2015). Experimental evidence of RIP – that is a mutagenic process targeted to duplicated DNA sequences that occurs during mating – is limited to fungi of the fungal classes Dothideomycetes [*L. maculans* (Idnurm and Howlett, 2003; Van de Wouw et al., 2019)] and Sordariomycetes [*Fusarium* spp., *Magnaporthe oryzae*, *Neurospora crassa*, *Podospora anserina*, and *Trichoderma reesei* (Selker and Garrett, 1988; Nakayashiki et al., 1999; Graïa et al., 2001; Cuomo et al., 2007; Coleman et al., 2009; Li et al., 2017), reviewed by (Hane et al., 2015)]. *In silico* sequence analysis suggests that RIP occurs extensively in the fungi [for example a potential activity in the Basidiomycota (Horns et al., 2012)], including species in the Eurotiales like *A. niger* (Braumann et al., 2008), *A. nidulans* (Nielsen et al., 2001; Clutterbuck, 2004), *Aspergillus oryzae* (Montiel et al., 2006), *Penicillium chrysogenum* (Braumann et al., 2008) and *Penicillium roqueforti* (Ropars et al., 2012). However, in these species whether those patterns of mutation represent RIP, the natural accumulation of mutations over time, or another mechanism of DNA mutation such as the spontaneous deamination of methylated cytosines (Lindahl, 1993; Lutsenko and Bhagwat, 1999), remains unknown. This point is well illustrated in the case of *A. nidulans*, a genetic model for many decades yet in which RIP has not been observed despite the *in silico* evidence (Nielsen et al., 2001; Clutterbuck, 2004). Second, we developed methods for the genetic transformation of *P. variotii*, including an efficient next-generation-sequencing-based method to identify genes that are mutated in forward genetic screens, and classical genetics in which parents are crossed and their progeny used in genetic segregation analysis. Using these new tools, we characterized two genes of previously unknown function.

By combining these methods, we demonstrate RIP activity experimentally for the first time in the Eurotiales, vastly expanding the phylogenetic breadth of the fungi experimentally verified to undergo RIP and thereby suggesting this is indeed a fundamental force that shapes fungal genome evolution. In addition, we compared the plant biomass degrading ability of *P. variotii* to other Eurotiales, hypothesizing that the active RIP mechanism in this species might reduce gene duplication events and thus limit the expansion of gene families in this species. Consistent with this hypothesis, our analysis revealed the poorest CAZy genome content in *P. variotii* among the fungal species used for comparison. This, and the identification of a phenotype associated with mutating a gene encoding a protein with a DUF1212 domain, which is at present an enigmatic yet widely conserved domain, highlights how research on *P. variotii* offers new perspectives to understand the biology of Eurotiales fungi, and fungi more broadly.

## Materials and Methods

### Wild-Type Strains and Preparation of Growth Media

The ex-type strain of *Paecilomyces variotii*, i.e., strain CBS 101075, was obtained from the Commonwealth Scientific and Industrial Research Organisation culture collection (FRR5219). A second strain was isolated as a contaminant after water damage to the laboratory, having attracted attention because of its ability to inhibit the growth of a plant pathogenic fungus *Leptosphaeria maculans*. This strain has been deposited at the Westerdijk Institute as CBS 144490. As described below, CBS 10105 (*MAT1-1*) and CBS 144490 (*MAT1-2*) are of opposite mating type. An *Aspergillus niger* strain was isolated from an onion (identification including ITS sequencing, as GenBank MH605508), and used as source of DNA in molecular biology experiments. The strain was deposited to the Westerdijk Institute as CBS 144491. The strains of Eurotiales species used for carbon utilization profiling are given in Table 1.

**Table 1:** Additional fungal species and strains used in this study.

Species	Strain	Reference
**CAZy gene comparison**		
*Talaromyces marneffei*	ATCC 18224	Nierman et al., 2015
*Penicillium rubens*	Wisconsin 54-1255	van den Berg et al., 2008
*Penicillium subrubescens*	FBCC1632, CBS 132785	Peng et al., 2017
*Aspergillus wentii*	CBS 141173	de Vries et al., 2017
*Aspergillus glaucus*	CBS 516.65	de Vries et al., 2017
*Aspergillus clavatus*	NRRL1	Sherlock et al., 2012
*Aspergillus fumigatus*	Af293	Nierman et al., 2005
*Aspergillus terreus*	NIH 2624	Sherlock et al., 2012
*Aspergillus oryzae*	RIB40	Machida et al., 2005
*Aspergillus nidulans*	FGSC A4	Galagan et al., 2005
*Aspergillus niger*	ATCC 1015	Andersen et al., 2011
**Secondary metabolite cluster comparison**		
*Aspergillus aculeatinus*	CBS 121060	Vesth et al., 2018
*Aspergillus bombycis*	NRRL 26010	Moore et al., 2016
*Aspergillus calidoustus*	SF006504	Horn et al., 2016
*Aspergillus fijiensis*	CBS 313.89	Vesth et al., 2018
*Aspergillus homomorphus*	CBS 101889	Vesth et al., 2018
*Aspergillus ibericus*	CBS 121593	Vesth et al., 2018
*Aspergillus nidulans*	FGSC A4	Galagan et al., 2005
*Aspergillus uvarum*	CBS 121591	Vesth et al., 2018
*Penicillium griseofulvum*	PG3	Banani et al., 2016
*Penicillium steckii*	IBT 24891	Nielsen et al., 2017
*Penicillium subrubescens*	FBCC1632, CBS132785	Peng et al., 2017
*Thermoascus aurantiacus*	ATCC 26904	


Cleared and uncleared V8 juice was adjusted to pH 6 with NaOH, and used at 10% v/v for media. Potato dextrose agar (PDA) and potato dextrose broth were obtained commercially (Difco). Minimal medium was prepared using an adaptation of Sutter (1975), and consisted of per liter: 20 g glucose, 2 g asparagine, 5 g KH_2_PO_4_, 500 mg MgSO_4_, 28 mg CaCl_2_, 2 mg citric acid⋅H_2_O, 1.5 mg ferric sulfate, 1 mg ZnSO_4_⋅7H_2_O, 300 μg MnSO_4_⋅H_2_O, 50 μg CuSO_4_⋅5H_2_O, 50 μg molybdic acid. 1 mg/ml leucine was added when needed.

### Genome Sequencing of *P. variotii* Strains

Genomic DNA of the two strains was isolated as described previously (Pitkin et al., 1996). The genome of *P. variotii* strain CBS 101075 was sequenced using the Pacific Biosciences platform. Unamplified libraries were generated using Pacific Biosciences standard template preparation protocol for creating > 10 kb libraries. Five μg of gDNA was used to generate each library and the DNA was sheared using Covaris g-TUBEs to generate sheared fragments of > 10 kb in length. The sheared DNA fragments were then prepared using Pacific Biosciences SMRTbell template preparation kit, where the fragments were treated with DNA damage repair, had their ends repaired so that they were blunt-ended, and 5′ phosphorylated. Pacific Biosciences hairpin adapters were ligated to the fragments to create the SMRTbell template for sequencing. The SMRTbell templates were then purified using exonuclease treatments and size-selected using AMPure PB beads. PacBio Sequencing primer was then annealed to the SMRTbell template library and sequencing polymerase was bound to them using Sequel Binding kit 2.0. The prepared SMRTbell template libraries were then sequenced on a Pacific Biosystem’s Sequel sequencer using v3 sequencing primer, 1M v2 SMRT cells, and Version 2.0 sequencing chemistry with 1 × 360 sequencing movie run times.

The filtered PacBio sub-read data were assembled together with Falcon version 1.8.8^1^, improved with FinisherSC version 2.0 (Lam et al., 2015), and polished with Arrow version SMRTLink v.5.0.0.6792.^2^

To aid in gene predictions and annotation, the *P. variotii* transcriptome was sequenced with Illumina. To generate a diversity of transcripts, mycelia were cultured under four conditions for 4 days without shaking: at two temperatures (30°C and 37°C) and in 10% cleared V8 juice pH 6 and potato dextrose broth. RNA was isolated from mycelium using TRIzol reagent (Invitrogen) following the manufacturer’s recommendations, and equal quantities of RNA isolated from each mycelium were pooled. Stranded cDNA libraries were generated using the Illumina Truseq Stranded RNA LT kit. mRNA was purified from 1 μg of total RNA using magnetic beads containing poly-T oligos. mRNA was fragmented and reverse transcribed using random hexamers and Superscript II (Invitrogen) followed by second strand synthesis. The fragmented cDNA was treated with end-pair, A-tailing, adapter ligation, and 8 cycles of PCR. The prepared library was then quantified using KAPA Biosystem’s next-generation sequencing library qPCR kit and run on a Roche LightCycler 480 real-time PCR instrument. The quantified library was then multiplexed with other libraries, and the pool of libraries was prepared for sequencing on the Illumina HiSeq sequencing platform utilizing a TruSeq paired-end cluster kit, v4, and Illumina’s cBot instrument to generate a clustered flow cell. Sequencing of the flow cell was performed on the Illumina HiSeq 2500 sequencer using HiSeq TruSeq SBS sequencing kits, v4, following a 2 × 150 indexed run recipe. Illumina reads were filtered for quality and artifacts, RNA spike-in, PhiX, and N-containing reads, trimmed and assembled into consensus sequences using Trinity version 2.3.2 (Grabherr et al., 2011).

The genome was annotated using the JGI Annotation Pipeline, and made publicly available via JGI fungal genome portal MycoCosm (Grigoriev et al., 2014).

Strain CBS 144490 HYG1 is a transformant of strain CBS 144490, with a T-DNA inserted into its genome from plasmid pCSB1. This strain was sequenced to represent the genome of CBS 144490. Illumina sequencing of strain CBS 144490 HYG1 was conducted at the Australian Genome Research Facility (AGRF), with 100 bp paired-end reads on an Illumina HiSeq 2500 instrument. The nuclear genome was assembled using Velvet with the k-mer setting at 67 and auto detect for low coverage cut off (Zerbino and Birney, 2008). The mitochondrial genome was assembled using the inbuilt assembler in Geneious version 10.1.3 and annotated along with the mitochondrial genome of strain CBS 101075 using MFannot (Supplementary Figure S1).

### Phylogenetic Analysis of Strains of *Paecilomyces*

Three gene regions, encoding calmodulin and β-tubulin and the internal transcribed spacers (ITS), were used to build phylogenetic trees between strains. Sequences were those used previously (Samson et al., 2009), with the addition of the corresponding regions of the “*P. variotii*” number 5 CBS 144490 and CBS 101075 obtained via BLAST searches of the whole genome sequences. Sequences were aligned using MUSCLE (Edgar, 2004) and phylogenetic relationships were inferred using MrBayes (Huelsenbeck and Ronquist, 2001) implemented through Geneious version 11.0.4 using the HKY85 substitution model and 1,100,000 iterations with the sequences from *Paecilomyces divaricatus* CBS 284.48 set as the outgroups.

In addition, a species tree was generated using 3,374 single copy gene orthologs, identified using mcl (Enright et al., 2002). Genes were individually aligned using mafft (Katoh and Standley, 2013), trimmed using Gblocks (Castresana, 2000) using the following parameters: -t = p, -e = .gb, and -b4 = 5, then the phylogeny was reconstructed using RAxML (Stamatakis, 2014) under the PROTGAMMAWAGF substitution model. 100 bootstrap replicates were performed (all branches were fully supported).

### Generation of Plasmids for Fungal Transformation Using *Agrobacterium tumefaciens*

Plasmids were constructed for the transformation of *P. variotii* using *A. tumefaciens* for differing purposes. These plasmids are described in the following eight subsections.

(i) Mitochondrial GFP barcode series. The nucleotide sequence corresponding to the first 76 amino acids of the *L. maculans* citrate synthase gene (*Lema_T101280.1*) was amplified using primers AU268 and AU269 (Supplementary Table S1) off the genomic DNA of strain M1. Suelmann and Fischer (2000) showed that the corresponding sequence from *A. nidulans* was sufficient to direct GFP localization to the mitochondria. The coding region of the GFP gene was amplified using primers AU108 and AU68 off plasmid PLAU17 (Idnurm et al., 2017). These two fragments were then cloned into plasmid PLAU2 (Idnurm et al., 2017) using Gibson assembly (New England Biolabs). The resultant plasmid was linearized with PmeI and a 20 nucleotide “barcode” was inserted into this site using Gibson assembly. The barcode contained 20 semi-randomized nucleotides (NMNMNMNMNMNMNMNMNMNM; where N is any nucleotide and M is either A or C, as based on Hensel et al. (1995), and appropriate flanking sequence for Gibson assembly was included as a single stranded oligonucleotide AU257 that was made double-stranded via a PCR reaction with primers AU258 and AU259. The pool of resulting fragments was cloned into the PmeI site resulting in a series of plasmids with different barcodes. The sequences of clones in individual plasmids were determined by Sanger sequencing with primer ai076 (Supplementary Table S2).

(ii) H2B-CFP. A fusion protein of *A. nidulans* histone H2B and GFP has previously been shown to be nuclear-localized (Maruyama et al., 2002). The coding region of the histone H2B gene of *A. niger* strain CBS 144491 was amplified using primers AU492 and AU493 off genomic DNA. The coding region of CFP was amplified using primers AU494 and AU495 off plasmid PLAU41 (a PLAU2-based expression plasmid for CFP, analogous to PLAU17) and cloned into the BglII site of PLAU2 using Gibson assembly.

(iii) *dspA* complementation construct. The *dspA* gene region was amplified using primers AU463 and AU464 and cloned into the XbaI site of plasmid pMAI2 (Idnurm et al., 2017) using Gibson assembly.

(iv) *prmJ* complementation construct. Two fragments corresponding to the gene were amplified with primers AU461 and AU438, and AU437 and AU462 and cloned into the XbaI site of plasmid pMAI2 (Idnurm et al., 2017).

(v) *A. niger prmJ* cross-species complementation construct. The coding region of the *A. niger prmJ* gene was amplified in two parts; the first using primers FD1212AFPLAU2 and FD1212ER, and FD1212DF and FD1212FRPLAU2 and then combined into the BglII site of PLAU53 (Idnurm et al., 2017) using Gibson assembly.

(vi) mCherry-tagged DspA. The coding region of the *dspA* gene was amplified by PCR using the *dspA* complementation construct as a template with primers AU516 and AU473. The mCherry coding sequence was amplified using primers AU474 and AU517. These two fragments were cloned into the BglII site of plasmid PLAU2 using Gibson assembly.

(vii) Mitochondrial GFP in a plasmid conferring resistance to G418. A plasmid expressing mitochondrially localized GFP and G418 resistance was created for co-localization experiments. The coding region of the citrate synthase-GFP fusion was amplified from plasmid CSB1 using primers AU268 and AU68 and cloned into the BglII site of plasmid PLAU53 (Idnurm et al., 2017) using Gibson assembly.

(viii) *leuA* gene knockout and complementation. A genomic fragment (1,449 bp) corresponding to the 5′ flank of the *leuA* homolog was amplified from strain CBS 101075 using primers MAI0442 and MAI0443. The 3′ flank of the gene (1,439 bp) was amplified with primers MAI0444 and MAI0445. The hygromycin expression cassette of plasmid pMAI17 was amplified using primers MAI0440 and MAI0441. The three fragments were cloned, using Gibson assembly, into plasmid pPZP-201BK (Covert et al., 2001) that had been linearized with EcoRI and HindIII restriction enzymes. *P. variotii* transformants were generated with this plasmid, as described below, and assayed for their ability to grow on minimal media without leucine. PCR analysis to confirm the successful integration of the knockout construct into the *leuA* gene was conducted using primer pairs: MAI0023 + MAI0446 and MAI0022 + MAI0447 that amplify from the *hph* gene into the 5′ or 3′ flank of the *leuA* locus, respectively.

As a complementation control, the wild type copy of *leuA* was amplified with primers MAI0442 and MAI0445 and cloned into pPZP-201BK linearized with EcoRI and HindIII. The plasmid and the empty pPZP-201BK were electroporated separately into *A. tumefaciens* strain EHA105. These two *A. tumefaciens* strains were co-cultured with two *leuA*Δ strains of *P. variotii* for 3 days, then overlaid with minimal medium and cefotaxime.

### Confirmation of T-DNA Insertion Sites and Verification of Complementation by PCR

The T-DNA insertion sites of two mutant strains were confirmed by PCR, i.e., strains AU2_33 and AU1_63. Primers used for AU2_33 were AU446 and ai076 for the intragenic T-DNA and Match2F and Match2R for the intergenic T-DNA. Primers used for 1_63 were AU437 and ai076. The integration of the constructs into the genome, for the complementation of strains, was confirmed by PCR using primers AU446 and AU448 for AU2_33 (*dspA*) and AU437 and AU439 for AU1_63 (*prmJ*).

### Transformation of *P. variotii* by *A. tumefaciens*

*Agrobacterium tumefaciens* strain EHA105 was transformed with plasmids by electroporation, as described previously (Urquhart and Idnurm, 2017), with selection on LB agar + 50 μg/ml kanamycin. An amount of *Agrobacterium* cells equivalent to a rice grain was scraped directly off the *Agrobacterium* transformation plate and suspended in 1 ml of SOC media. *P. variotii* spores were harvested off V8 agar cultures and suspended in dH_2_O at approximately 10^6^ spores per ml. Five hundred μl of fungal spores and 100 μl of *Agrobacterium* suspensions were pipetted onto the center of a 145 mm petri dish containing 25 ml of solidified induction media (Gardiner and Howlett, 2004). The mixture was spread around the plate and incubated at 22°C for 3 days and then overlaid with 25 ml of molten CV8 containing 200 μg/ml cefotaxime and either 100 μg/ml hygromycin or 200 μg/ml G418 as appropriate for selection of transformants. Leucine (10 mg/ml) was added to the overlay in the case of the transformation aiming at gene replacement of *leuA*. Fungal transformants appeared after 5 days and were transferred onto fresh V8 plates containing half the antibiotic concentrations used in the overlay.

### Barcoding Mutagenesis and NGS to Locate DNA Inserts

DNA was extracted from a number of *P. variotii* transformants that showed growth phenotypes, using a buffer containing CTAB as described previously (Pitkin et al., 1996). The genomic DNA was pooled and sequenced at the AGRF with Illumina sequencing using the same instrument and parameters as strain CBS 144490. Analysis of the next generation sequencing data was conducted in Geneious version 10.1.2. To identify the positions of T-DNA insertions in the genome of a given strain the NGS reads containing the “barcode” from the construct with which that strain was transformed were pulled out (Supplementary Figure S2). Many of these reads extended out from the T-DNA into the sequence adjacent to the T-DNA and this section of the *P. variotii* genome was then identified using BLAST against the genome sequence.

### Microscopy

A Leica M205 stereomicroscope was used for the examination of mating cultures on agar plates. Fluorescence microscopy was performed using a Leica DM6000 microscope. Cell wall staining was conducted using calcofluor white M2R (0.0004%) and emission was detected using a DAPI filter cube. Images were overlaid using ImageJ software.

### Genetic Crosses

Crossing was conducted as described by Houbraken et al. (2008). Recombination in the progeny was confirmed using genetic markers that were based on PCR amplification of genomic fragments followed by digestion with restriction enzymes. An exception was for the mating type locus where a multiplex PCR resulting in different product sizes was employed. These markers and primers used for amplification are summarized in Supplementary Table S3.

### Amplification and Sequencing of the *hph* Gene Conferring Hygromycin Resistance From Progeny of a AU2_33 × CBS 101075 Cross

A region of each of the T-DNAs was amplified using primer MAI0022 located at the start of the hygromycin phosphotransferase (*hph*) open reading frame and a primer specific to the genomic region flanking each of the T-DNA insertion sites (primer Match2R or primer AU439). The resulting PCR product was then used as the template from which to amplify the *hph* coding region by PCR using primers MAI0022 and MAI0023. This PCR product was sequenced using Sanger chemistry at the AGRF.

### Southern Blot Analysis of T-DNA Insert Copy Number

Approximately 10 μg of genomic DNA was digested with HindIII and separated on a 1% agarose gel by electrophoresis. DNA was blotted onto Hybond-N+ membrane (GE Healthcare) using standard methods. A fragment of the *hph* gene was labeled with the PCR DIG Probe Synthesis Kit (Roche), as per the manufacturer’s directions, hybridized to the blot overnight, and the probe was detected using the DIG wash and block buffer set (Roche) and the DIG Luminescent Detection Kit following the manufacturer’s directions. An image of the blot was captured with a ChemiDoc MP (Bio-Rad) using the High Sensitivity Chemi setting.

### Profiling Fungal Growth on Different Carbon Sources

Fungi were grown on *Aspergillus* minimal medium containing 25 mM monosaccharide or 1% polysaccharide for 2–5 days (depending on the species; Table 1), after which pictures were taken. Growth was compared using D-glucose as an internal reference, so that growth on a specific carbon source relative to growth on glucose was compared between the species.

### Analysis of Gene Content

Gene numbers in different functional categories for the two *P. variotii* strains were obtained using the “cluster” option from the MycoCosm portal (Grigoriev et al., 2014), comparing the two strains with other species in the Eurotiales as well as *Neurospora crassa* where RIP is most extensively characterized. As a focused case study, the putative Carbohydrate-Active enzymes (CAZys) were filtered for families known to be involved in plant biomass degradation (de Vries et al., 2017). It should be noted that for families the genes could not always be split by the predicted activity, resulting in some cases in an over-prediction of the number of genes encoding enzymes for the utilization of a certain polysaccharide.

## Results

### Genome Sequence Characteristics of *P. variotii* Strains CBS 101075 and CBS 144490

The genome of ex-type strain *P. variotii* CBS 101075 was sequenced using long reads of Pacific Biosciences technology, and genes were annotated using the JGI annotation pipeline (Grigoriev et al., 2014). The mitochondrial genome was annotated separately with MFannot software (Supplementary Figure S1). The *P. variotii* CBS 101075 genome is approximately 30.1 Mb in total size, 4.53% of which is comprised of repetitive DNA of simple repeats and putative transposable elements (Supplementary Table S4). The genome appears to represent gene-encoding regions completely, as estimated by the presence of 100% of CEGMA genes [the Core Eukaryotic Genes Mapping Approach (Parra et al., 2007)]. Analysis using Benchmarking Universal Single-Copy Orthologs (BUSCO V3; Waterhouse et al., 2018) also indicated a high level of completeness to the genome, with 99.6 and 99.0% of BUSCO genes being present using the Fungi or Eukaryota settings, respectively. Assembly statistics are comparable to other related, recently published genomes (Supplementary Table S4). We identified 9,270 genes in the *P. variotii* genome (Supplementary Table S4), most of which are complete by having both start and stop codons (98.76%) and have well supported matches in various genomic databases, including NCBI (95.2% of genes) and Pfam (75.02%) (Finn et al., 2016). MCL-based ortholog clustering (Enright et al., 2002) using the genomes in Supplementary Table S4 reveals 8,808 *P. variotii* genes in orthologous gene clusters, and 462 unique genes. The genome assembly and related data for *P. variotii* CBS 101075 is available from https://genome.jgi.doe.gov/Paevar1, and the whole genome shotgun project deposited to GenBank as accession RCNU00000000.

The genome of a second isolate, CBS 144490 HYG1, was generated using short read technology. A total of 15,229,380 100 bp paired end reads were generated and were assembled into 126 contigs (N50 = 642,740) totaling 32,365,222 bp. This genome was annotated based on that of CBS 101075, and is available from https://genome.jgi.doe.gov/Paevar_HGY_1, and deposited in GenBank under accession RHLL00000000 and in the short read archive as PRJNA497137.

### Phylogenetic Resolution of Sequenced Strains Within the *Paecilomyces* Genus

A phylogenetic analysis was conducted to confirm the species-level taxonomy of strain CBS 144490, and “*P. variotii*” strain number 5 whose genome was previously sequenced (Oka et al., 2014). The calmodulin and β-tubulin gene regions and ITS separates *P. formosus* and *P. variotii* into separate clades (Supplementary Figure S3), in agreement with previous studies (Samson et al., 2009). The regions obtained from the genome sequence of CBS 101075 were identical to those deposited previously for this isolate in GenBank. Strain CBS 144490, isolated in this study, also clearly groups with the other *P. variotii* strains. However, strain “*P. variotii*” number 5 (Oka et al., 2014) groups within the *P. formosus* clade, and not with *P. variotii*.

### *Agrobacterium tumefaciens* Can Be Used for the Efficient Transformation of *P. variotii*

**FIGURE 1:**
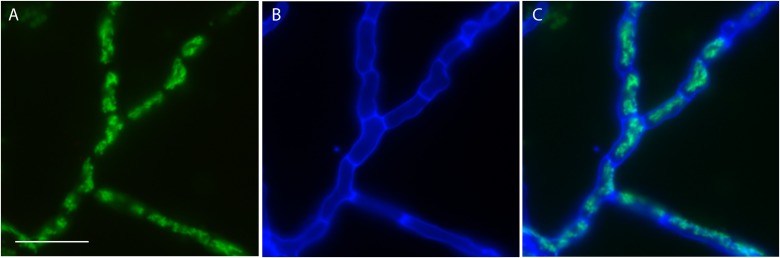
Transformation of *P. variotii* using *Agrobacterium*-mediated delivery of the exogenous DNA. The construct encodes an enzyme conferring hygromycin resistance for selection and a hybrid protein with a mitochondrial-targeted sequence fused to GFP. **(A)** Mitochondrial GFP fluorescence of was observed in 100 out of 100 hygromycin-resistant strains obtained after transformation; one representative strain is shown. **(B)** Cell walls fluoresce blue from staining with calcofluor white. **(C)** The overlay of the GFP and calcofluor white signals. Scale bar = 20 μm.

Although the genomes of *P. variotii* contain a number of interesting genes and other features, testing their function requires methods for genetic manipulation. In the first step for this process, transformation with exogenous DNA was tested using delivery of T-DNA molecules from *Agrobacterium tumefaciens*. The T-DNA used expressed hygromycin phosphotransferase, GFP with an N-terminal mitochondrial targeting sequence, and contained a “barcode” sequence inward of the right border. Following selection on hygromycin, colonies were examined for GFP fluorescence: the hyphae of all strains (*n* = 100) had fluorescent tubules consistent with mitochondria, indicating that when using this transformation system 100% of the resultant colonies have integrated the T-DNA construct, including the DNA for expression of GFP, into their genome (Figure 1).

### Targeted Gene Disruption in *P. variotii* Is Possible Despite the Multinucleate Nature of Its Conidiospores

**FIGURE 2:**
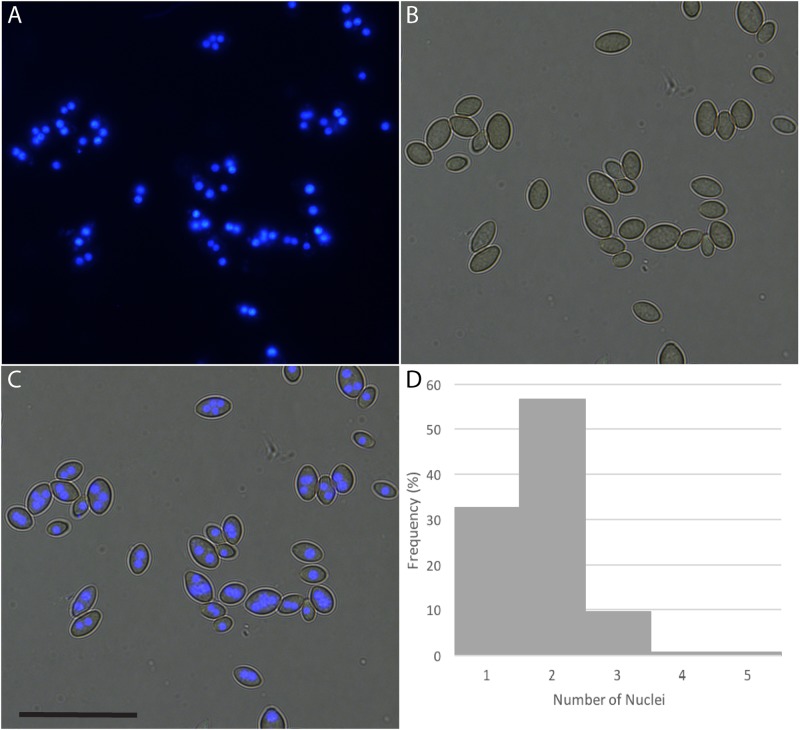
*Paecilomyces variotii* produces a mix of uni- and multinucleate spores. Nucleus copy numbers in strain CBS 101075 conidiospores were visualized through the expression of a CFP-Histone H2B fusion construct. **(A)** CFP fluorescence, **(B)** DIC image, and **(C)** overlay, scale bar = 25 μm. **(D)** Histogram of the distribution of the number of nuclei per spore (*n* = 138); two nuclei per spore is the most common, and 32.6% of spores are uninucleate.

Many fungi produce multinucleate spores, meaning that after transformation several passaging steps are required to isolate a homokaryotic mycelium. A histone H2B-CFP fusion construct, causing the localization of CFP to the nucleus, was transformed into *P. variotii* to allow the number of nuclei in the conidiospores to be counted. Most of the spores contained two or more nuclei and some spores containing up to four nuclei (Figure 2).

**FIGURE 3:**
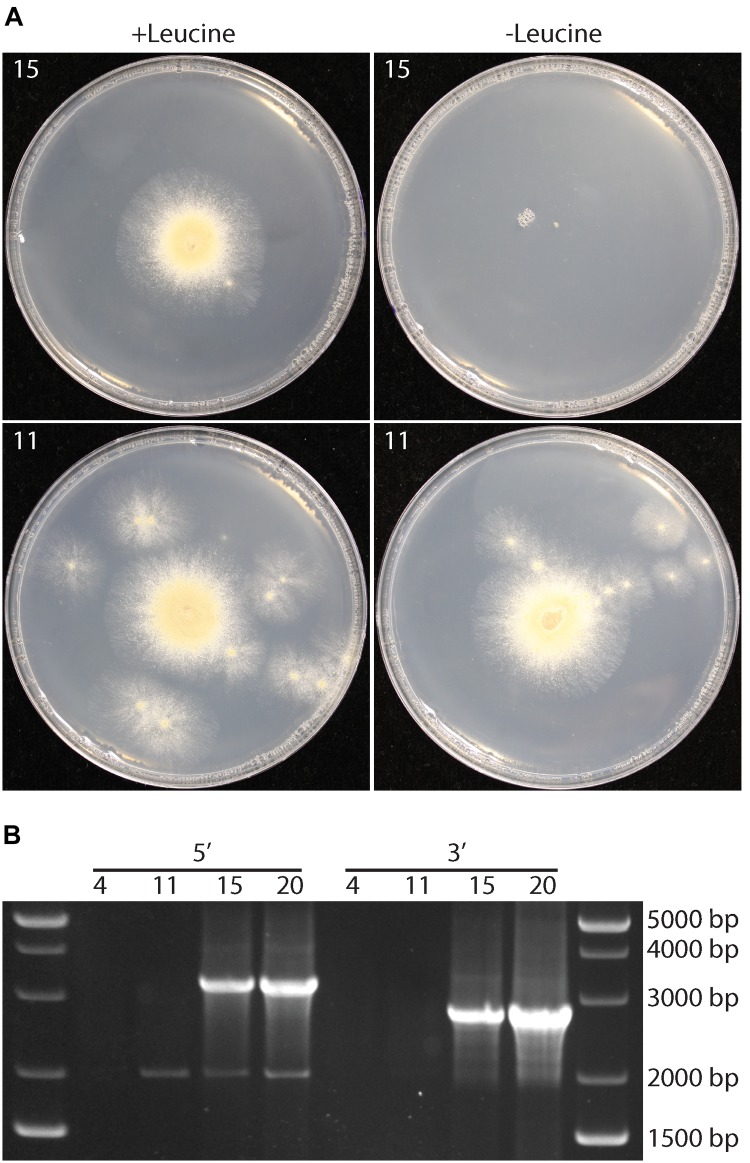
Targeted gene disruption, through homologous recombination, of the *leuA* gene in *P. variotii*. **(A)** Growth of two representative transformants, 15 that is a putative *leuA* deletion strain and 11 that is an ectopic insertion of the deletion construct, on minimal medium with (+) or without (–) leucine. **(B)** PCR amplification of the 5′ and 3′ regions adjacent to *leuA* into the *hph* selectable marker gene illustrate correct integration of the knockout construct by amplifying sequences unique to a correct integrant (3,440 and 2,850 bp) in transformants 15 and 20, but not in ectopic insertion strains 4 and 11.

The experiments above indicated that *P. variotii* could be transformed with DNA. However, whether targeted gene mutations were possible and if mutants could be easily isolated from a population containing multinucleate spores were unknown. To address this, the feasibility of targeted gene disruption via homologous recombination in this species was tested. The *leuA* gene, encoding α-isopropylmalate synthase required for leucine biosynthesis, was chosen as mutation of homologs of the gene results in leucine auxotrophy in ascomycetes, basidiomycetes and Mucoromycota species (Kohlhaw, 2003; Larson and Idnurm, 2010; Ianiri et al., 2011) that are easy to identify by their inability to growth on media without leucine. Of 25 hygromycin-resistant strains transformed with the *leuA* knockout construct, which contains approximately 1.5 kb of homologous sequence on either side of the construct for hygromycin resistance, four showed reduced growth on minimal media without amino acids and 21 showed wild type growth rate (Figure 3A). The growth of the four strains was restored by the addition of leucine to the medium (Figure 3A). PCR analysis confirmed the correct integration of the hygromycin resistance cassette into the *leuA* locus in the four leucine auxotrophs (Figure 3B).

To confirm that the leucine auxotrophy was due to the gene deletion, two deletion strains were complemented with the wild type copy of *leuA*. The full length gene was amplified from wild type DNA and cloned into plasmid pPZP-201BK. The pPZP-201BK-leuA and pPZP-201BK empty plasmids were used to transform the two strains using *Agrobacterium*-mediated delivery of their T-DNAs, with selection on minimal medium without leucine. Colonies were obtained when using the *leuA* plasmid, but not empty plasmid (data not shown).

### Rapid Identification of T-DNA Insertion Sites in Barcoded Mutants by Next-Generation Sequencing

To assess the potential for forward genetics using insertional mutagenesis of T-DNA molecules delivered from *Agrobacterium* in *P. variotii*, approximately 500 transformants were screened for growth or development phenotypes on V8 juice medium and minimal medium. Transformants with such phenotypes were obtained, and seven were further investigated toward identifying the genes disrupted within them.

A NGS approach was used in which a pool of DNA from the seven strains carrying a barcode near the right border of the T-DNA was sequenced, to identify the location of T-DNA insertion sites (Supplementary Table S5). Three of the strains were found to each contain at least two T-DNA insertions. No reads containing barcode number 4 could be found and thus the location of the T-DNA is strain AU4_W could not be determined. Three of the strains contained the same barcode sequence (barcode 1). Only two T-DNAs corresponding to barcode 1 were found. However, reads were present which contained barcode 1 and vector sequence extending beyond the right border, so it is likely that one of these strains contains an abnormally integrated T-DNA. Two of the strains in which the T-DNA had clearly inserted within the open reading frame of genes were further studied, namely strains AU1_63 and AU2_33.

**FIGURE 4:**
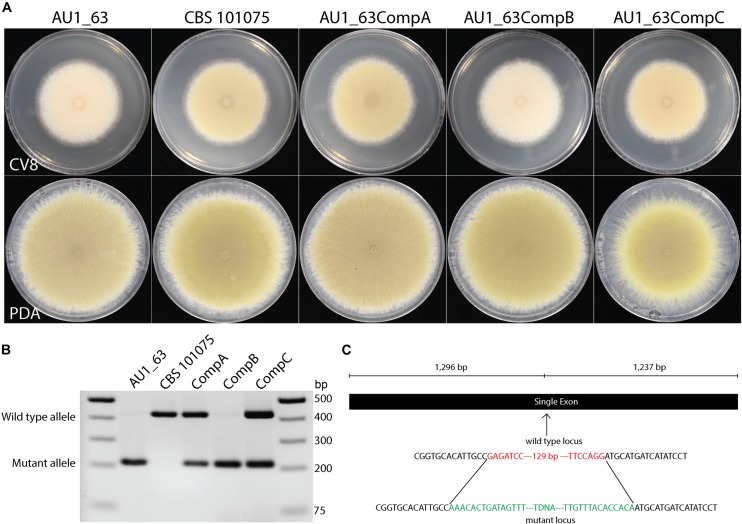
**(A)** Mutation of the *prmJ* gene, in strain AU1_63, results in a pale phenotype on cleared V8 juice (CV8) agar but not on potato dextrose agar (PDA). Two of the three strains transformed with the complementation construct have a wild type phenotype while one resembles the *prmJ* mutant. **(B)** PCR analysis of the genotypes of the AU1_63 mutant, wild type CBS 101075, and the three strains after transformation with the complementation construct. The AU1_63CompB transformant, in which the phenotype was not complemented, has not integrated a wild-type copy of the *prmJ* gene. **(C)** Location of the T-DNA insert in the *prmJ* gene. Green represents sequence of the T-DNA and red represents nucleotides lost when the T-DNA integrated into the genome in the mutant strain AU1_63.

Three of the strains whose DNA were pooled for sequencing were derived from transformation with the same plasmid so therefore contained the same barcode sequence (#1). PCR was employed to distinguish the insertion events between them, to reveal the presence of the mutation in a gene with a domain of unknown function (DUF1212) in strain AU1_63 (Figure 4). The insert is located approximately in the center of the single exon of the gene, upstream of the region encoding the conserved DUF1212 domain (Figure 4C). We named this gene *prmJ* after the *Saccharomyces cerevisiae* homolog *PRM10*, employing the gene nomenclature used for *A. nidulans* and other Eurotiales species to *P. variotii*.

**FIGURE 5:**
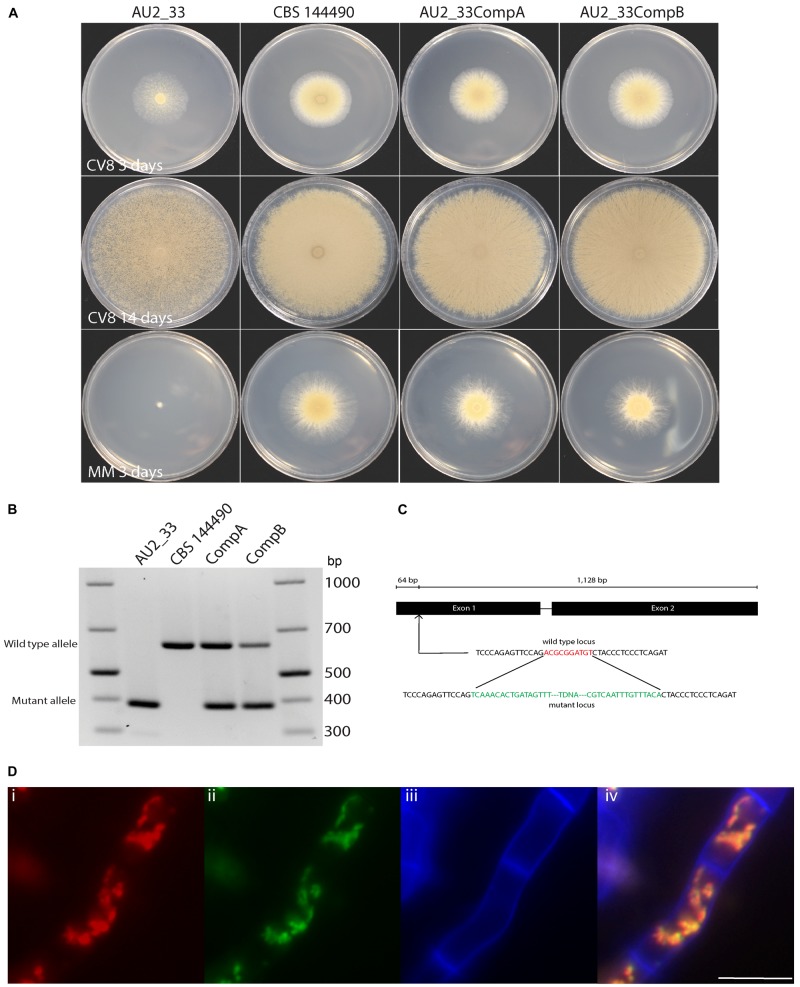
Strain AU2_33 has a growth and sporulation defect due to mutation of the *dspA* gene. **(A)** Sporulation on CV8 was delayed in the mutant AU2_33 at both 3 and 14 days after growth on clear V8 juice medium compared to the wild type CBS 144490 and two complemented strains. The AU2_33 mutant also had impaired growth on minimal medium (MM). **(B)** PCR analysis of the genotypes of the AU2_33 mutant, wild type and two complemented isolates. **(C)** The T-DNA insertion is located in the first exon of the *dspA* gene. Green represents sequence of the T-DNA and red represents sequence lost from the genome in the mutant. **(D)** Co-localization of mCherry-tagged DspA protein and mitochondrially localized GFP: **(i)** red fluorescence from the DspA-mCherry fusion, **(ii)** green fluorescence of citrate synthase-GFP, **(iii)** blue fluorescence due to calcofluor white staining of the cell wall, and **(iv)** the merged image. Scale bar = 10 μm.

The strain AU2_33 contains two insertion sites, one in the coding region of a mitochondrial membrane carrier (delayed sporulation A, *dspA*) and one that was intergenic. The genes near the intergenic insertion were not further characterized. The intragenic T-DNA insert was located 64 bp into the first exon of the *dspA* gene (Figure 5C).

Strains AU1_63 and AU2_33 were analyzed by Southern blotting to confirm the number of T-DNA inserts as indicated by the genome sequencing data (Supplementary Figure S4). The single T-DNA insertion in strain AU1_63 was supported by the *hph* gene fragment hybridizing to a single HindIII fragment of approximately 3.9 kb, while two T-DNAs in strain AU2_33 were indicated as hybridization to two HindIII restriction fragments of ∼6.4 kb and ∼8.3 kb. These sizes are consistent with size predictions based on HindIII sites in the genome sequence data adjacent to the T-DNA insertion sites.

### Strain AU1_63 Has a Media-Dependent Impairment in Spore Pigmentation, Due to Mutation of a Gene With an Uncharacterized Domain

Strain AU1_63 has a pale phenotype on cleared V8 juice (CV8) agar medium because it produces conidiospores that lacked the characteristic yellow pigmentation of *P. variotii* (Figure 4A). The phenotype was not observed when the strain was cultured instead on potato dextrose medium. A wild type copy of the *prmJ* gene was amplified and cloned into a plasmid containing a construct conferring G418 resistance, and then transformed into strain AU1_63. Of three strains transformed with the complementation construct, two had a phenotype similar to wild type and one resembled the AU1_63 mutant. However, PCR analysis showed that this non-complementing transformant has not integrated the wild-type copy of the gene into its genome whereas the two other strains with the wild type spore pigmentation had (Figure 4B).

*Paecilomyces variotii* is heterothallic, and comparison of strains CBS 144490 (*MAT1-1*) and CBS 101075 (*MAT1-2*) revealed that each has a distinct gene complement at its *MAT* locus (Supplementary Figure S5). The pair therefore allows the potential for crossing. The 32 progeny of a cross between mutant AU1_63 and CBS 144490 showed prefect co-segregation of hygromycin resistance with the pale colony pigmentation, as shown in Table S6. Two additional genetic markers, 123A and 123B (Supplementary Table S3), located 1,069,000 bp apart on contig 123 of CBS 144490 were examined in these progeny. These markers demonstrated that recombination events take place during crossing, consistent with meiotic reduction events rather than parasexual reduction in chromosome numbers as can occur in some Eurotiales species.

### The Mutation in *prmJ* in Strain AU1_63 Can Be Cross-Species Complemented by the *Aspergillus niger prmJ* Homolog

The DUF1212-containing protein (PrmJ) identified in *P. variotii* shows strong sequence similarity to homologs from the genus *Aspergillus*. As a representative example, the alignment of the *A. niger* homolog (GenBank: EHA28452.1) has 66% identical amino acids with PrmJ of *P. variotii*.

**FIGURE 6:**
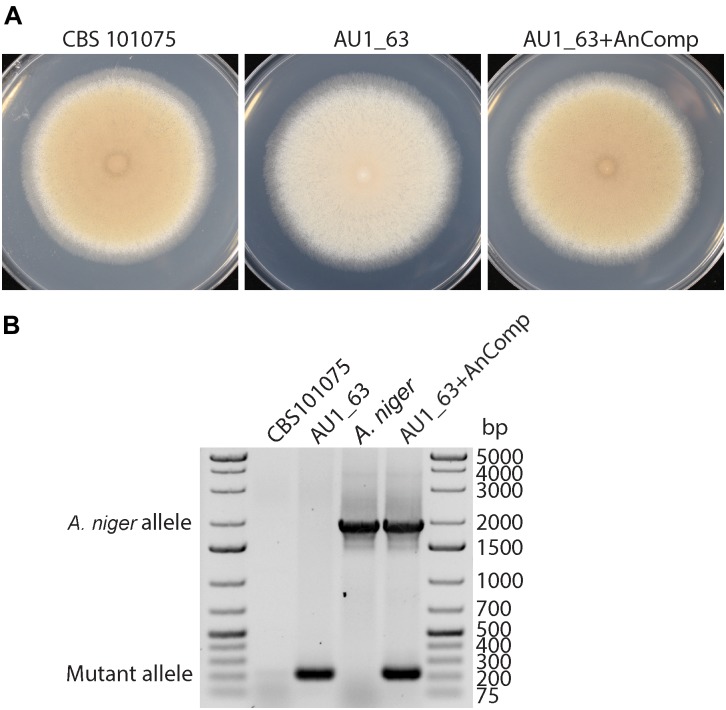
Cross-species complementation of the *P. variotii* AU1_63 strain with the *Aspergillus niger prmJ* homolog restores the colony pigmentation to wild type levels of the AU1_63 mutant of CV8 media. **(A)** Growth of the wild type (CBS 101075), T-DNA insertion mutant (AU1_63), and insertion mutant transformed with the *A. niger prmJ* gene (AU1_63+AnComp) on cleared V8 juice medium. **(B)** A PCR analysis for the *P. variotii* mutated allele and the introduced *A. niger* alleles in the three strains, with wild type *A. niger* as a control.

To test the hypothesis that the PrmJ proteins have a conserved function, the coding region of the homologous gene from *A. niger* was cloned into the constitutive expression plasmid PLAU2 and transformed into the AU1_63 mutant. Five putative transformants were obtained; all showed an increase in colony pigmentation, and one of these transformants was further analyzed (Figure 6A). PCR confirmed that the transformant contained both the mutated copy of the *prmJ* allele and the introduced *A. niger* transgene (Figure 6B). Thus, the *A. niger* homolog can complement the functions lost in the *P. variotii prmJ* gene mutant.

### The DUF1212 Domain Protein Is Not Essential for Mating in *P. variotii*

There is little information about DUF1212 proteins in fungi, other than that the *PRM10* gene of *S. cerevisiae* is transcriptionally induced in response to sexual pheromones (Heiman and Walter, 2000). Of the progeny of the AU1_63 × CBS 101075 cross, eight contained the disrupted *prmJ* allele and were of the opposite mating type (*MAT1-2*) to strain AU1_63 (*MAT1-1*) (Supplementary Table S6). One of these isolates was back-crossed to strain AU1_63, and this combination of strains was able to produce the sexual cleistothecia structures and viable progeny from ascospores (Supplementary Figure S6). Hence, the DUF1212 domain protein is not essential for sexual crossing in *P. variotii*.

### Strain AU2_33 Has Delayed Sporulation and Growth Defect Phenotypes Due to Mutation of the Mitochondrial Membrane Carrier DspA

Strain AU2_33 showed delayed sporulation on CV8 medium, with spore production beginning at around 3–4 days, in contrast to the wild type that produces spores as soon as the colony begins to expand (Figure 5A). Even after 14 days, the amount of sporulation was reduced. On this medium the radial growth rate was not noticeably reduced. In contrast, on defined minimal medium, the radial growth rate of the AU2_33 mutant was highly reduced as it showed close to no growth. A complementation construct was produced with a wild type copy of the gene, and when transformed into strain AU2_33, the transformants showed a phenotype resembling that of the wild type. As expected, PCR analysis of the two complemented isolates indicated that they contain both a mutant and a wild-type allele in their genomes (Figure 5B).

Transformants of CBS 101075 expressing a DspA-mCherry fusion protein displayed red fluorescence. This co-localized with the green fluorescence of a mitochondrially localized GFP-citrate synthase fusion protein, indicating that this putative carrier protein also localizes to the mitochondrion (Figure 5D).

The T-DNA insertion in strain AU2_33 co-segregated with the delayed sporulation phenotype in 18 out of 20 progeny as assessed by PCR (Supplementary Table S7). Two progeny, 17 and 19, contained the mutant *dspA* allele yet did not display the mutant phenotype, which might be due to the effect of other genetic rearrangements taking place during crossing. The two T-DNA inserts of mutant AU2_33 displayed genetic linkage co-segregating in 19 of 20 progeny. There was recombination between the T-DNAs and mating type locus, demonstrating the progeny were the result of meiotic events. Intriguingly, all of the progeny from this cross were sensitive to hygromycin.

### The *hph* Gene, Conferring Hygromycin Resistance, Is Mutated by Repeat-Induced Point Mutation (RIP) in the Progeny of a Cross Between AU2_33 and CBS 101075

**FIGURE 7:**
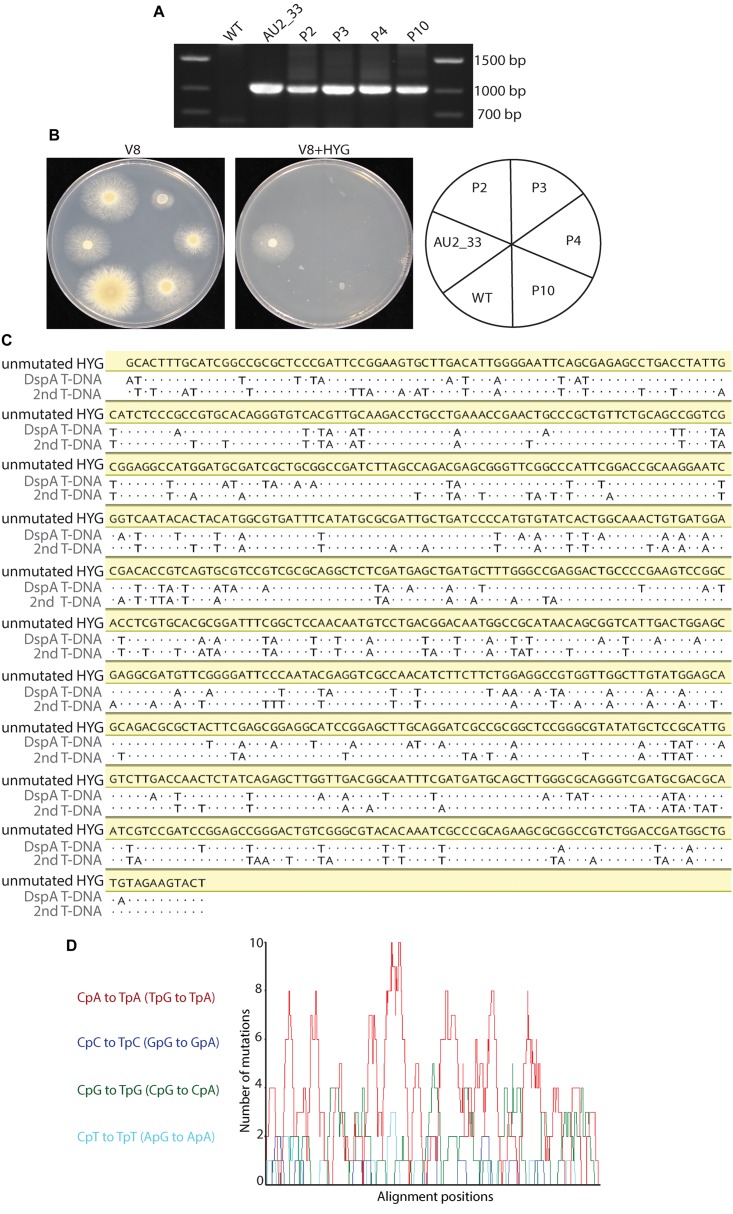
Repeat induced point mutation is active in the Eurotiales. **(A)** PCR analysis indicates that four progeny (P2, P3, P4, and P10) of a cross between the wild type (WT) and AU2_33 contain the hygromycin phosphotransferase gene, despite **(B)** these progeny being sensitive to hygromycin (HYG). **(C)** Nucleotide sequence alignment of 780 bp of the *hph* coding region of both T-DNAs in progeny number 3 showed a pattern of C to T and A to G mutations, consistent with the RIP process. Dots represent identical nucleotides. **(D)** RIPCAL analysis of the sequencing information in **(C)** revealed a basis towards mutation of CpA dinucleotides, also consistent with RIP mutation.

None of the 20 progeny resulting from a cross between mutant AU2_33 and CBS 101075 showed a hygromycin resistance phenotype, despite the T-DNA construct being present in 10 of these progeny as demonstrated by PCR analysis (Figures 7A,B). Therefore the coding region of the *hph* gene, which confers resistance to hygromycin, in one of the progeny (progeny 3) was sequenced. The open reading frame of the *hph* gene amplified from both T-DNA insertion copies revealed substitution mutations characteristic of RIP (Figure 7C). A 780 bp region was sequenced and 141 (18.1%) and 156 (20%) nucleotides were mutated. The mutations were all C to T or G to A. RIPCAL analysis revealed bias toward CpA to TpA dinucleotides and the complementary TpG to TpA mutations that are characteristic of RIP in other fungal species such as *N. crassa* (Figure 7D).

### The *P. variotii* Genome Features Evidence of RIP

**FIGURE 8:**
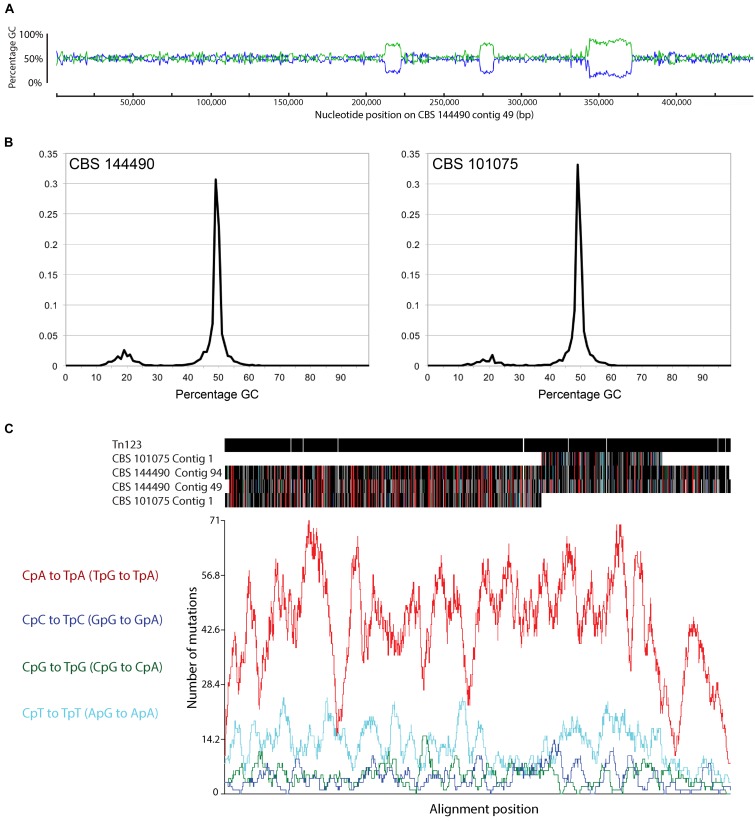
**(A)** *Paecilomyces variotii* isolates show a bipartite genome structure that is characteristic of a consequence of repeat induced point mutation acting in the organism. **(B)** The genome assembly of strain CBS 144490 contains a greater proportion of AT-rich regions than does that of strain CBS 101075 **(C)**. A putative transposon was identified on contig 123 of CBS 144490 with similarity to some of the AT-rich regions present in both genomes: RIPCAL analysis revealed that most of the putative mutations were CpA to TpA (TpG to TpA in the reverse strand), which is a feature of DNA that has undergone RIP.

The genome sequences of *P. variotii* have a bimodal GC content, containing long stretches of approximately 50% G:C interspersed by relatively shorter regions of approximately 20% G:C. The example of the first 450,000 bp of CBS 144490 contig 49 is given in Figure 8A. Overall, these AT rich regions constitute approximately 8.49% of the CBS 101075 assembly and 13.8% of CBS 144490 assembly (Figure 8B). It should be noted that because of the different sequencing strategies – long reads from Pacific Bioscience vs. 100 nucleotide reads from Illumina technologies – these proportions can only be compared broadly. One mechanism by which AT-rich regions can be created is RIP (Testa et al., 2016), which has been shown to defend the *N. crassa* genome against transposons (Kinsey et al., 1994). We hypothesize that the AT-rich regions identified in the *P. variotii* genome are due to RIP.

In support of this hypothesis, a putative Tf2-type retrotransposon, Tn123, on contig 123 (nucleotide position 231,587–238,677) of CBS 144490 was identified via BLASTx searches (Altschul et al., 1990). BLASTn comparison of this sequence against the two *P. variotii* genomes revealed sequence similarity between this transposon and a number of the AT-rich regions in both genomes. Furthermore, there was a clear pattern of C → T and G → A mutations that are characteristic of RIP. RIPCAL analysis showed that most of the RIP-like mutations targeted CpA dinucleotides, which is also highly characteristic of RIP [(Hane et al., 2015); Figure 8C]. This strongly suggested that RIP mutation of retrotransposons including, but not limited to Tn123, is responsible for the formation of at least some of these AT-rich regions.

### *P. variotii* Has a Reduced Expansion of Gene Families, Including Polysaccharide Degradation Related CAZy Genes, Relative to Other Eurotiales Species

**FIGURE 9:**
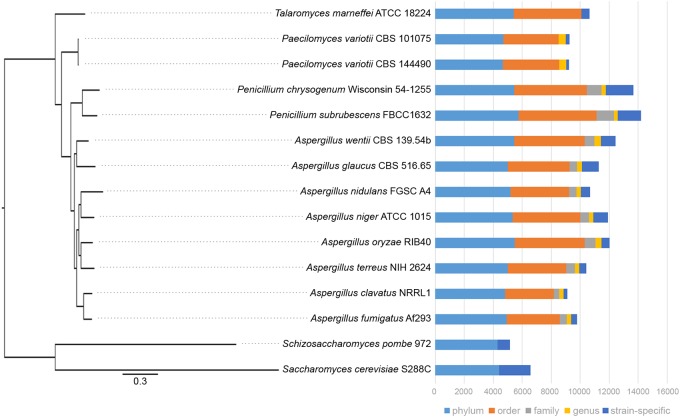
*Paecilomyces variotii* has fewer genes than many other species in the Eurotiales. Phylogenetic relationships between the Eurotiales species, with two yeast species as outgroups, were defined from a comparison of 3,374 single copy gene orthologs. The graph shows total numbers of genes in each species and the distribution of the homologs.

**FIGURE 10:**
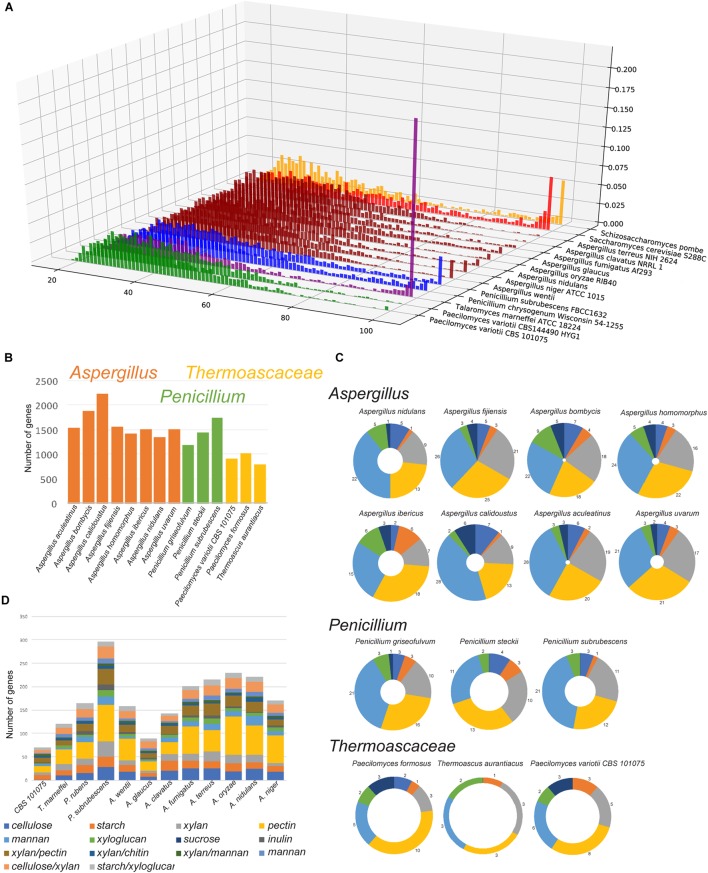
*Paecilomyces variotii* and related Thermoascaceae have a reduced expansion in gene families. **(A)** A limited number of highly similar gene duplicates are observed in *P. variotii* compared to other Eurotiales. For each genome, a self BLASTp was conducted to identify orthologs by reciprocal best hit via BLAST, then the fraction of orthologs at various identity levels were plotted. *x*-axis: percent identity, *y*-axis: lineage, *z*-axis: fraction of all orthologs at a given % identity. Lineages are colored at the genus level, green: *Paecilomyces*, purple: *Talaromyces*, blue: *Penicillium*, dark red: *Aspergillus*, red: *Saccharomyces*, yellow: *Schizosaccharomyces*. **(B)** Three Thermoascaceae species have relatively fewer genes in the 100 most populous gene clusters in the comparative cluster analysis obtained through MycoCosm. Similarly, these species showed a more restricted set of **(C)** secondary metabolite genes and **(D)** genes encoding Carbohydrate-Active enZYmes (CAZys).

Given the genomic and experimental evidence for the active occurrence of RIP in *P. variotii* we assessed whether a consequence is the limited expansion of gene families in this species. A comparison of *P. variotii* with other Eurotiales species shows that these strains have the fewest genes (Figure 9). We compared the bi-directional similarity of *P. variotii* genes against the second closest BLAST match in its own genome. Consistent with RIP *P. variotii* has fewer genes with close similarity than the comparison species in the genera *Talaromyces*, *Penicillium*, *Saccharomyces*, *Schizosaccharomyces* and most *Aspergilli*. However, several *Aspergillus* species including *A. clavatus* also had few similar genes, possibly indicating past or current RIP in these species (Figure 10A).

Comparative cluster data obtained through the MycoCosm portal (Grigoriev et al., 2014) show that *P. variotii* along with two other species in the family Thermoascaceae (*P. formosus* and *Thermoascus aurantiacus*) contains considerably fewer genes in the 100 most populous gene clusters (Figure 10B and Supplementary Table S8). For example, examination of secondary metabolite gene clusters shows that the three species in the Thermoascaceae contain fewer secondary metabolite clusters than other species in the Eurotiales (Figure 10C). *P. variotii* is particularly depleted in genes encoding polyketide synthases, with strain CBS 101075 containing only six such genes compared to as many as 28 in some of the *Aspergillus* species examined. Two other striking reductions in gene family numbers were seen for amino acid permeases (cluster 12) and major facilitator superfamilies (clusters 10 and 13). The one exception to the reduction in gene numbers in the Thermoascaceae species examined was an expansion in genes encoding methyltransferases (cluster 11).

Comparison of the CBS 144490 genome with that of CBS 101075 revealed a high level of similarity; however, CBS 144490 contains an additional 1.2 Mb of sequence, some of which is made up of repetitive elements, while estimated to have 40 fewer genes overall (Supplementary Table S4). A comparison between genomes revealed that the CBS 144490 strain has 372 genes and the CBS 101075 strain has 450 genes that are unique to each strain and not found in the other. No examples of recent DNA duplications were observed in either genome. In many cases, genes unique to one or the other strain were found as clusters of varying size of such unique genes. The most striking example is the presence of scaffold 108 (151 kb) in CBS 144490 that is absent from CBS 101075. This region includes 52 predicted genes, including a putative non-ribosomal peptide synthase. However, despite these differences to date no *in vitro* growth differences have been observed for the two strains.

Evidence for the lack of expansion of gene families in *P. variotii* can be seen in the genes encoding CAZys (for plant polysaccharide degradation), as *P. variotii* had the fewest number of such genes (74 genes) of all tested Eurotiales species (Figure 10D and Supplementary Dataset S1). In total, this number is most similar to *Aspergillus glaucus* (92 genes), while significantly higher CAZy gene numbers in all of the other species suggests a better capability for plant polysaccharide degradation.

### Assimilation Capabilities Are Reduced in *P. variotii* for Some Carbohydrate Sources

**FIGURE 11:**
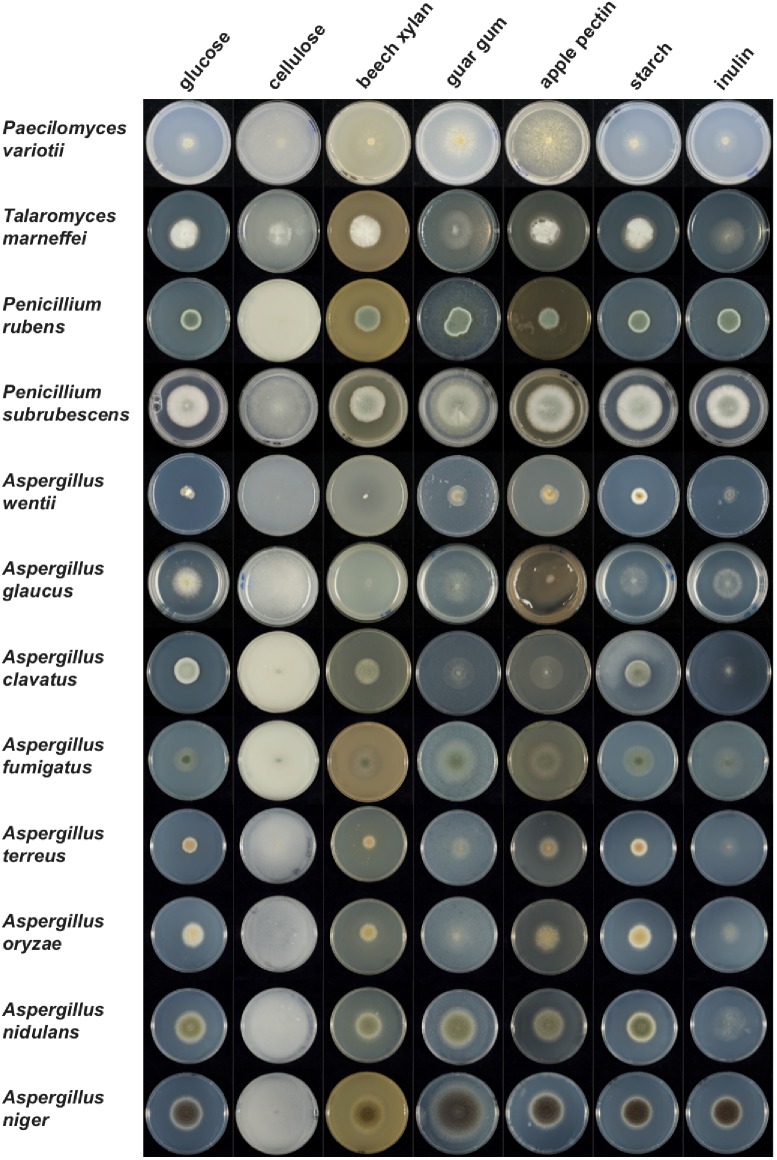
Growth of *P. variotii* compared to other Eurotiales species on different plant polysaccharides as the sole carbon source, compared to glucose. Petri dishes containing minimal medium and differing carbon sources were inoculated with different species of Eurotiales and growth photographed after 2–5 days depending on the species.

To assess if the reduction in gene family numbers has a consequence on biology, the growth of *P. variotii* on different carbon sources was compared with other Eurotiales species. Overall, *P. variotii* is less able to use these plant-derived compounds as a carbon source than most other species (Figure 11). The growth profile of *P. variotii* is also most similar to *A. glaucus*, consistent with the genome content of CAZys.

Growth of *P. variotii* is particularly poor on cellulose, xylan and inulin, which correlates with the very low number of genes encoding cellulolytic (8 genes), xylanolytic (30 genes) and inulinolytic (2 genes) enzymes compared to other species in the Eurotiales. *Talaromyces marneffei* has no inulinolytic genes and *A. nidulans* has the same number as *P. variotii*: these species also grow very poorly on inulin, as do several others (Supplementary Dataset S1 and Figure 11). Interestingly, *P. variotii* can produce high levels of invertase when cultivated on agricultural and industrial residues (Job et al., 2010). Growth on commercial cellulose (Avicel) is challenging for most fungi, so it is hard to use it to draw comparative conclusions about species differences, although reasonable radial growth was observed for *P. variotii*. However, *P. variotii* has been shown to produce a glucose-tolerant β-glucosidase (Job et al., 2010) indicating its ability to release glucose molecules from short oligosaccharides. A clear difference in growth of the species is seen on xylan, but this does not fully correlate with the number of xylanolytic genes. Growth is poor for *P. variotii* and *A. glaucus* that have a low number of xylanolytic genes, but also for *A. wentii* that has a similar number (66 genes) to species that grow well on this substrate. In contrast, good growth was observed for *T. marneffei* (47 genes), which has a similar number of genes as *A. glaucus* (41 genes). However, a *P. variotii* strain showing high xylanase production has been described (de Laguna et al., 2015), suggesting that even with a few genes those enzymatic activities may reach high levels.

*Paecilomyces variotii* grows relatively well on guar gum (galactomannan) even though it has a low number of mannanolytic genes in its genome (10 genes), similar to *A. glaucus*. Both species grow better on guar gum than on xylan, suggesting that their limited enzyme system is sufficient for degradation of galactomannan. Neither species contains a known endomannanase, but both contain exo-enzymes, β-mannosidases and α-galactosidases that can release the monomeric sugars from galactomannan. *A. niger* and *P. subrubescens*, which both have a much more extensive mannanolytic gene system, including several endomannanase encoding genes, grow much better on guar gum.

Similarly to guar gum, *P. variotii* showed good growth on apple pectin despite having the lowest number of pectinolytic genes (25 genes) from the tested species. Exo-polygalacturonases have also been purified from *P. variotii* cultures further demonstrating its pectinolytic capability (de Lima Damásio et al., 2010; Patil et al., 2012). This is in contrast to poor growth of *A. clavatus*, which has a reduced number of pectinolytic genes (43 genes) compared to most other Aspergilli, but still almost twice as many as *P. variotii*. The better growth of *P. variotii* on apple pectin could be explained by a higher number of GH28 pectin hydrolases (6 genes) compared to *A. clavatus* (3 genes). This may also explain the poorer growth of *A. glaucus* on this substrate, as while it has a similar number of pectinolytic genes as *P. variotii*, it only contains two genes encoding GH28 enzymes.

*Paecilomyces variotii* has been shown to produce thermostable glucoamylase and α-amylase with potential in industrial applications (Michelin et al., 2008; Michelin et al., 2010). Growth of *P. variotii* on starch was similar to most other species. Despite a somewhat reduced amylolytic gene set (16 genes), *P. variotii* has all the enzymatic activities for degradation of starch, which likely explains the growth observed on this polysaccharide.

## Discussion

The genus *Paecilomyces* has received limited attention for functional genomics, despite its role in industry, human disease, and as a commonly encountered saprobe found around the world. This research has generated high quality genome sequence resources, demonstrates that genetic segregation analysis is possible, and shows that gene disruption by either targeted or reverse genetics is highly feasible for gene discovery. Several key points arising from this work are described in the following sections.

Using *P. variotii*, new properties associated with fungal genes of unknown function have been defined. For example, proteins with a DUF1212 are widely conserved in fungi and include Prm10 in *S. cerevisiae* and NCU00717 in *N. crassa*. In *S. cerevisiae*, the gene was found to be up-regulated three-fold in response to pheromone and predicted to contain five transmembrane segments (Heiman and Walter, 2000). However, the biological function of these proteins has not been elucidated and no phenotypes found in gene disruption strains. This study reports a pigmentation phenotype associated with disruption of the DUF1212 homolog in *P. variotii* (Figure 4). Given that the *PRM10* gene is up-regulated in response to pheromone in *S. cerevisiae* (Heiman and Walter, 2000), we assessed whether the protein is required for mating in *P. variotii*. Crosses between two isolates carrying the DUF1212 mutant allele produced cleistothecia, sexual spores and viable progeny (Supplementary Figure S6). This suggests that the *PRM10* homolog (*prmJ*) is not essential for the sexual cycle of *P. variotii*. The ability of the *A. niger prmJ* homolog to complement the pigmentation phenotype of the *P. variotii* mutant strain implies that the function of this protein is conserved between the two genera (Figure 6). Identification of a phenotype associated with a Domain of Unknown Function (DUF) protein that has a conserved function in a related species suggests that *P. variotii* is a species in which to study this protein family in greater detail.

A second insertional mutant investigated in detail contained a T-DNA in the *dspA* gene encoding a mitochondrial carrier family protein. DspA is localized to the mitochondria and mutation of the *dspA* gene delays sporulation in a manner dependent on the medium composition (Figure 5). As in the case of the AU1_63 strain, the phenotype of the AU2_33 (*dspA* mutant) strain is influenced by the composition of the media. On a minimal medium, growth on the strain was highly restricted (Figure 5). This provides a possible direction for future studies into the function of this putative mitochondrial carrier protein. That is, if a compound can be found that when supplemented into the media restores the phenotype of this mutant, that compound might represent the substrate of the carrier. Despite their annotation, not all mitochondrial carrier family proteins are localized to the mitochondria. For example, proteins in this family have been found localized to chloroplasts (Palmieri et al., 2009) and peroxisomes (Jank et al., 1993). Thus, the localization of mitochondrial carrier family proteins cannot be predicted, so must be determined experimentally. We demonstrate, through co-localization with a known mitochondrial protein, that the DspA protein in *P. variotii* has a mitochondrial localization.

An unexpected finding from the genetic segregation analysis of mutant AU2_33, which contains two T-DNAs, was that all of the progeny were hygromycin sensitive despite half of the progeny encoding the hygromycin resistance gene when they were tested by PCR (Figure 7). We traced this loss of resistance to mutation of the *hph* gene by RIP. Thus, this analysis in *P. variotii* provides important evidence that Eurotiales fungi have active RIP mechanisms.

Analysis of the *P. variotii* genome sequence shows evidence for past RIP activity, both in its bi-modal G:C content and more conclusively the presence of a putative retrotransposon, Tn123, some copies of which strongly appear to have been affected by RIP (Figure 8). In the majority of genomes analyzed for past RIP activity there is a dinucleotide profile that is biased toward RIP-like CpA mutations (Hane et al., 2015). Analysis of the Tn123 sequences also indicated a strong CpA bias, strengthening our conclusion that the mutated copies of this transposon sequence have been created through RIP mutation (Figure 8).

The predicted consequences of RIP are limitations in the expansion of genes by gene duplication. Evidence for this comes from the analysis of gene families when compared with other ascomycete species. As illustrated in Figures 9, 10 and Supplementary Table S8, *P. variotii* consistently has the lowest number of genes other than *N. crassa*, where RIP has been demonstrated to occur, and *Thermoascus aurantiacus*, where little is know about its genetics. While one predicted consequence of RIP should be limitation in the expansion of gene families, this is not always the case: in this analysis the species with the largest number of families, *Nectria haematococca*, also has an active RIP process (Coleman et al., 2009). Analysis of the ability of *P. variotii* to degrade polysaccharides suggests that it has much smaller gene set related to the degradation of plant polysaccharides compared to most of the other tested Eurotiales species (Figure 10D). One interpretation of this finding is that RIP mutation has reduced gene duplication and thus the expansion of these gene families. This may represent evidence of the hypothesized evolutionary cost associated with the genome protection afforded by RIP (Galagan and Selker, 2004). A flipside of the evolutionary cost of RIP has also been hypothesized that certain loci within repeat rich compartments may undergo accelerated evolution; this remains to be experimentally validated.

Despite recent advances, not least in the rapid rate of genome sequencing (Grigoriev et al., 2014), only a minute fraction of the millions of fungal species believed to exist (Blackwell, 2011) have been studied at the genetic level. This is because the necessary combination of tools required for functional biology, i.e., a genome sequence, transformation protocols, targeted gene mutations and genetic crosses, have been developed in relatively few species. However, research conducted beyond the current model organisms is vital to gain a more comprehensive understanding of fungal biology.

*Paecilomyces variotii* is one of the vast number of fungal species for which techniques for genetic manipulation have not previously been reported, despite its relevance to human activities. In this study, we have produced genome assemblies for two strains as well as developing transformation, efficient targeted gene disruption using *Agrobacterium* and convenient genetic crosses. Considering that PEG-mediated protoplast transformation is commonly used in several species of the Eurotiales (Nara et al., 1993; de Bekker et al., 2009; Arentshorst et al., 2012; Oakley et al., 2012; Weyda et al., 2017), it is likely that this protocol could also be adapted to *P. variotii*. Taken together, *P. variotii* could now be considered as a convenient model for studying aspects of the diverse biology of the Eurotiales (de Vries et al., 2017), and in particular the family Thermoascaceae, including studying RIP. Future work will undoubtedly uncover more novelties or shared features in this ubiquitous organism.

## Author Contributions

AU, SM, MM, AW, and AI performed the experiments. AU, SM, GH, SM, JP, AL, KB, IG, and AI were involved in genome sequencing, assembly and annotation. AU, SM, JH, IG, and AI analyzed the data. AU, MM, SM, RdV, IG, and AI designed the experiments, discussed the results, and wrote the manuscript. All authors provided final approval for the manuscript.

## Conflict of Interest Statement

The authors declare that the research was conducted in the absence of any commercial or financial relationships that could be construed as a potential conflict of interest.

## Footnotes

1 https://github.com/PacificBiosciences/FALCON

2 https://github.com/PacificBiosciences/GenomicConsensus

## References

[B1] Altschul S. F. Gish W. Miller W. Myers E. W. Lipman D. J. (1990). Basic local alignment search tool. *J. Mol. Biol.* 215 403–410. 10.1016/S0022-2836(05)80360-2 2231712

[B2] Andersen M. R. Salazar M. P. Schaap P. J. van de Vondervoort P. J. I. Culley D. Thykaer J. (2011). Comparative genomics of citric-acid-producing *Aspergillus niger* ATCC 1015 versus enzyme-producing CBS 513.88. *Genome Res.* 21 885–897. 10.1101/gr.112169.110 21543515 PMC3106321

[B3] Arentshorst M. Ram A. F. J. Meyer V. (2012). Using non-homologous end-joining-deficient strains for functional gene analyses in filamentous fungi. *Methods Mol. Biol.* 835 133–150. 10.1007/978-1-61779-501-5-9 22183652

[B4] Banani H. Marcet-Houben M. Ballester A. R. Abbruscato P. González-Candelas L. Gabaldón T. (2016). Genome sequencing and secondary metabolism of the postharvest pathogen *Penicillium griseofulvum*. *BMC Genomics* 17:19. 10.1186/s12864-015-2347-x 26729047 PMC4700700

[B5] Battestin V. Macedo G. A. (2007a). Purification and biochemical characterization of tannase from a newly isolated strain of *Paecilomyces variotii*. *Food Biotechnol.* 21 207–216. 10.1080/08905430701533588

[B6] Battestin V. Macedo G. A. (2007b). Tannase production by *Paecilomyces variotii*. *Bioresour. Technol.* 98 1832–1837. 10.1016/j.biortech.2006.06.031 17045475

[B7] Bellanger A. P. Cervoni J. P. Faucher J. F. Weil-Verhoeven D. Ginet M. Deconinck E. (2017). *Paecilomyces variotii* fungemia in a patient with lymphoma needing liver transplant. *Mycopathologia* 182 761–765. 10.1007/s11046-017-0131-y 28365835

[B8] Biango-Daniels M. N. Wang T. W. Hodge K. T. (2018). Draft genome sequence of the patulin-producing fungus *Paecilomyces niveus* strain CO7. *Genome Announc.* 6:e00556–18. 10.1128/genomeA.00556-18 29930063 PMC6013598

[B9] Blackwell M. (2011). The fungi: 1,2, 3. 5.1 million species? *Am. J. Bot.* 98 426–438. 10.3732/ajb.1000298 21613136

[B10] Braumann I. van den Berg M. Kempken F. (2008). Repeat induced point mutation in two asexual fungi, *Aspergillus niger* and *Penicillium chrysogenum*. *Curr. Genet.* 53 287–297. 10.1007/s00294-008-0185-y 18347798

[B11] Castresana J. (2000). Selection of conserved blocks from multiple alignments for their use in phylogenetic analysis. *Mol. Biol. Evol.* 17 540–552. 10.1093/oxfordjournals.molbev.a026334 10742046

[B12] Clutterbuck A. J. (2004). MATE transposable elements in *Aspergillus nidulans*: evidence of repeat-induced point mutation. *Fungal Genet. Biol.* 41 308–316. 10.1016/j.fgb.2003.11.004 14761791

[B13] Coleman J. J. Rounsley S. D. Rodriguez-Carres M. Kuo A. Wasmann C. C. Grimwood J. (2009). The genome of *Nectria haematococca*: contribution of supernumerary chromosomes to gene expansion. *PLoS Genet.* 5:e1000618. 10.1371/journal.pgen.1000618 19714214 PMC2725324

[B14] Covert S. F. Kapoor P. Lee M.-H. Briley A. Nairn C. J. (2001). *Agrobacterium tumefaciens*-mediated transformation of *Fusarium circinatum*. *Mycol. Res.* 105 259–264. 10.1017/S0953756201003872

[B15] Cuomo C. A. Güldener U. Xu J.-R. Trail F. Turgeon B. G. Di Pietro A. (2007). The *Fusarium graminearum* genome reveals a link between localized polymorphism and pathogen specialization. *Science* 317 1400–1402. 10.1126/science.1143708 17823352

[B16] de Bekker C. Wiebenga A. Aguilar G. Wösten H. A. B. (2009). An enzyme cocktail for efficient protoplast formation in *Aspergillus niger*. *J. Microbiol. Methods* 76 305–306. 10.1016/j.mimet.2008.11.001 19041907

[B17] de Laguna I. H. B. Marante F. J. T. Mioso R. (2015). Enzymes and bioproducts produced by the ascomycete fungus *Paecilomyces variotii*. *J. Appl. Microbiol.* 119 1455–1466. 10.1111/jam.12934 26274842

[B18] de Lima Damásio A. R. da Silva T. M. Maller A. Jorge J. A. Terenzi H. F. de Moraes Polizeli M. D. L. T. (2010). Purification and partial characterization of an exo-polygalacturonase from *Paecilomyces variotii* liquid cultures. *Appl. Biochem. Biotechnol.* 160 1496–1507. 10.1007/s12010-009-8682-0 19484410

[B19] de Vries R. P. Riley R. Wiebenga A. Aguilar-Osorio G. Amillis S. Uchima C. A. (2017). Comparative genomics reveals high biological diversity and specific adaptations in the industrially and medically important fungal genus *Aspergillus*. *Genome Biol.* 18:28. 10.1186/s13059-017-1151-0 28196534 PMC5307856

[B20] Edgar R. C. (2004). MUSCLE: multiple sequence alignment with high accuracy and high throughput. *Nucleic Acids Res.* 32 1792–1797. 10.1093/nar/gkh340 15034147 PMC390337

[B21] Enright A. J. Van Dongen S. Ouzounis C. A. (2002). An efficient algorithm for large-scale detection of protein families. *Nucleic Acids Res.* 30 1575–1584. 10.1093/nar/30.7.1575 11917018 PMC101833

[B22] Feldman R. Cockerham L. Buchan B. W. Lu Z. Huang A. M. (2016). Treatment of *Paecilomyces variotii* pneumonia with posaconazole: case report and literature review. *Mycoses* 59 746–750. 10.1111/myc.12525 27401982

[B23] Finn R. D. Coggill P. Eberhardt R. Y. Eddy S. R. Mistry J. Mitchell A. L. (2016). The Pfam protein families database: towards a more sustainable future. *Nucleic Acids Res.* 44 D279–D285. 10.1093/nar/gkv1344 26673716 PMC4702930

[B24] Galagan J. E. Calvo S. E. Cuomo C. Ma L.-J. Wortman J. R. Batzoglou S. (2005). Sequencing of *Aspergillus nidulans* and comparative analysis with *A. fumigatus* and *A. oryzae*. *Nature* 438 1105–1115. 16372000 10.1038/nature04341

[B25] Galagan J. E. Selker E. U. (2004). RIP: the evolutionary cost of genome defense. *Trends Genet.* 20 417–423. 10.1016/j.tig.2004.07.007 15313550

[B26] Gardiner D. M. Howlett B. J. (2004). Negative selection using thymidine kinase increases the efficiency of recovery of transformants with targeted genes in the filamentous fungus *Leptosphaeria maculans*. *Curr. Genet.* 45 249–255. 10.1007/s00294-004-0488-6 14749893

[B27] Grabherr M. G. Haas B. J. Yassour M. Levin J. Z. Thompson D. A. Amit I. (2011). Full-length transcriptome assembly from RNA-Seq data without a reference genome. *Nat. Biotechnol.* 29 644–652. 10.1038/nbt.1883 21572440 PMC3571712

[B28] Graïa F. Lespinet O. Rimbault B. Dequard-Chablat M. Coppin E. Picard M. (2001). Genome quality control: RIP (repeat-induced point mutation) comes to *Podospora*. *Mol. Microbiol.* 40 586–595. 10.1046/j.1365-2958.2001.02367.x 11359565

[B29] Grigoriev I. V. Nikitin R. Haridas S. Kuo A. Ohm R. Otillar R. (2014). MycoCosm portal: gearing up for 1000 fungal genomes. *Nucleic Acids Res.* 42 D699–D704. 10.1093/nar/gkt1183 24297253 PMC3965089

[B30] Hane J. K. Williams A. H. Taranto A. P. Solomon P. S. Oliver R. P. (2015). “Repeat-induced point mutation: a fungal-specific, endogenous mutagenesis process,” in *Genetic Transformation Systems in Fungi* Vol. 2 eds van den Berg M. A. Maruthachalam K. (New York, NY: Springer), 55–68.

[B31] Heidarian R. Fotouhifar K.-B. Debets A. J. B. Aanen D. K. (2018). Phylogeny of *Paecilomyces*, the causal agent of pistachio and some other trees dieback disease in Iran. *PLoS One* 13:e0200794. 10.1371/journal.pone.0200794 30040828 PMC6057626

[B32] Heiman M. G. Walter P. (2000). Prm1p, a pheromone-regulated multispanning membrane protein, facilitates plasma membrane fusion during yeast mating. *J. Cell Biol.* 151 719–730. 10.1083/jcb.151.3.719 11062271 PMC2185589

[B33] Hensel M. Shea J. E. Gleeson C. Jones M. D. Dalton E. Holden D. W. (1995). Simultaneous identification of bacterial virulence genes by negative selection. *Science* 269 400–403. 10.1126/science.7618105 7618105

[B34] Horn F. Linde J. Mattern D. J. Walther G. Guthke R. Scherlach K. (2016). Draft genome sequences of fungus *Aspergillus calidoustus*. *Genome Announc.* 4:e00102–16. 10.1128/genomeA.00102-16 26966204 PMC4786660

[B35] Horns F. Petit E. Yockteng R. Hood M. E. (2012). Patterns of repeat-induced point mutation in transposable elements of basidiomycete fungi. *Genome Biol. Evol.* 4 240–247. 10.1093/gbe/evs005 22250128 PMC3318451

[B36] Houbraken J. Samson R. A. Frisvad J. C. (2006). *Byssochlamys*: significance of heat resistance and mycotoxin production. *Adv. Exp. Med. Biol.* 571 211–224. 10.1007/0-387-28391-9_14 16408604

[B37] Houbraken J. Varga J. Rico-Munoz E. Johnson S. Samson R. A. (2008). Sexual reproduction as the cause of heat resistance in the food spoilage fungus *Byssochlamys spectabilis* (anamorph *Paecilomyces variotii*). *Appl. Environ. Microbiol.* 74 1613–1619. 10.1128/AEM.01761-07 18192427 PMC2258620

[B38] Houbraken J. Verweij P. E. Rijs A. J. M. M. Borman A. M. Samson R. A. (2010). Identification of *Paecilomyces variotii* in clinical samples and settings. *J. Clin. Microbiol.* 48 2754–2761. 10.1128/JCM.00764-10 20519470 PMC2916617

[B39] Huelsenbeck J. P. Ronquist F. (2001). MRBAYES: bayesian inference of phylogenetic trees. *Bioinformatics* 17 754–755. 10.1093/bioinformatics/17.8.754 11524383

[B40] Ianiri G. Wright S. A. I. Castoria R. Idnurm A. (2011). Development of resources for the analysis of gene function in *Pucciniomycotina* red yeasts. *Fungal Genet. Biol.* 48 685–695. 10.1016/j.fgb.2011.03.003 21402165

[B41] Idnurm A. Howlett B. J. (2003). Analysis of loss of pathogenicity mutants reveals that repeat-induced point mutations can occur in the Dothideomycete *Leptosphaeria maculans*. *Fungal Genet. Biol.* 39 31–37. 10.1016/S1087-1845(02)00588-1 12742061

[B42] Idnurm A. Urquhart A. S. Vummadi D. R. Chang S. Van de Wouw A. P. López-Ruiz F. J. (2017). Spontaneous and CRISPR/Cas9-induced mutation of the osmosensor histidine kinase of the canola pathogen *Leptosphaeria maculans*. *Fungal Biol. Biotechnol.* 4:12. 10.1186/s40694-017-0043-0 29270298 PMC5732519

[B43] Jank B. Habermann B. Schweyen R. J. Link T. A. (1993). PMP47, a peroxisomal homolog of mitochondrial solute carrier proteins. *Trends Biochem. Sci.* 18 427–428. 8291088

[B44] Job J. Sukumaran R. K. Jayachandran K. (2010). Production of a highly glucose tolerant β-glucosidase by *Paecilomyces variotii* MG3: optimization of fermentation conditions using Plackett–Burman and Box–Behnken experimental designs. *World J. Microbiol. Biotechnol.* 26 1385–1391. 10.1007/s11274-010-0311-0

[B45] Katoh K. Standley D. M. (2013). MAFFT multiple sequence alignment software version 7: improvements in performance and usability. *Mol. Biol. Evol.* 30 772–780. 10.1093/molbev/mst010 23329690 PMC3603318

[B46] Kinsey J. A. Garrett-Engele P. W. Cambareri E. B. Selker E. U. (1994). The *Neurospora* transposon *Tad is* sensitive to repeat-induced point mutation (RIP). *Genetics* 138 657–664. 7851763 10.1093/genetics/138.3.657 PMC1206216

[B47] Kohlhaw G. B. (2003). Leucine biosynthesis in fungi: entering metabolism through the back door. *Microbiol. Mol. Biol. Rev.* 67 1–15. 10.1128/MMBR.67.1.1-15.2003 12626680 PMC150519

[B48] Kondo T. Morikawa Y. Hayashi N. (2008). Purification and characterization of alcohol oxidase from *Paecilomyces variotii* isolated as a formaldehyde-resistant fungus. *Appl. Microbiol. Biotechnol.* 77 995–1002. 10.1007/s00253-007-1237-9 17985128

[B49] Kuboi T. Okazaki K. Inotani M. Sugino M. Sadamura T. Nakano A. (2016). A case of cutaneous *Paecilomyces formosus* infection in an extremely premature infant. *J. Infect. Chemother.* 22 339–341. 10.1016/j.jiac.2015.12.003 26774294

[B50] Lam K. K. Labutti K. Khalak A. Tse D. (2015). FinisherSC: a repeat-aware tool for upgrading de novo assembly using long reads. *Bioinformatics* 31 3207–3209. 10.1093/bioinformatics/btv280 26040454

[B51] Larson E. M. Idnurm A. (2010). Two origins for the gene encoding α-isopropylmalate synthase in fungi. *PLoS One* 5:10. 10.1371/journal.pone.0011605 20657649 PMC2904702

[B52] Li W.-C. Huang C.-H. Chen C.-L. Chuang Y.-C. Tung S.-Y. Wang T.-F. (2017). *Trichoderma reesei* complete genome sequence, repeat-induced point mutation, and partitioning of CAZyme gene clusters. *Biotechnol. Biofuels* 10:170. 10.1186/s13068-017-0825-x 28690679 PMC5496416

[B53] Lindahl T. (1993). Instability and decay of the primary structure of DNA. *Nature* 362 709–715. 10.1038/362709a0 8469282

[B54] Lutsenko E. Bhagwat A. S. (1999). Principal causes of hot spots for cytosine to thymine mutations at sites of cytosine methylation in growing cells – A model, its experimental support and implications. *Mutat. Res.* 437 11–20. 10.1016/S1383-5742(99)00065-4 10425387

[B55] Machida M. Asai K. Sano M. Tanaka T. Kumagai T. Terai G. (2005). Genome sequencing and analysis of *Aspergillus oryzae*. *Nature* 438 1157–1161. 10.1038/nature04300 16372010

[B56] Maruyama J. Nakajima H. Kitamoto K. (2002). Observation of EGFP-visualized nuclei and distribution of vacuoles in *Aspergillus oryzae arpA* null mutant. *FEMS Microbiol. Lett.* 206 57–61. 10.1111/j.1574-6968.2002.tb10986.x 11786257

[B57] Max B. Salgado J. M. Rodríguez N. Cortés S. Converti A. Domínguez J. M. (2010). Biotechnological production of citric acid. *Braz. J. Microbiol.* 41 862–875. 10.1590/S1517-83822010000400005 24031566 PMC3769771

[B58] Michelin M. Ruller R. Ward R. J. Moraes L. A. B. Jorge J. A. Terenzi H. F. (2008). Purification and biochemical characterization of a thermostable extracellular glucoamylase produced by the thermotolerant fungus *Paecilomyces variotii*. *J. Ind. Microbiol. Biotechnol.* 35 17–25. 10.1007/s10295-007-0261-1 17938981

[B59] Michelin M. Silva T. M. Benassi V. M. Peixoto-Nogueira S. C. Moraes L. A. B. Leão J. M. (2010). Purification and characterization of a thermostable α-amylase produced by the fungus *Paecilomyces variotii*. *Carbohydr. Res.* 345 2348–2353. 10.1016/j.carres.2010.08.013 20850111

[B60] Montiel M. D. Lee H. A. Archer D. B. (2006). Evidence of RIP (repeat-induced point mutation) in transposase sequences of *Aspergillus oryzae*. *Fungal Genet. Biol.* 43 439–445. 10.1016/j.fgb.2006.01.011 16531081

[B61] Moore G. G. Mack B. M. Beltz S. B. Gilbert M. K. (2016). Draft genome sequence of an aflatoxigenic *Aspergillus* species, *A. bombycis*. *Genome Biol. Evol.* 8 3297–3300. 10.1093/gbe/evw238 27664179 PMC5203779

[B62] Nakayashiki H. Nishimoto N. Ikeda K. Tosa Y. Mayama S. (1999). Degenerate MAGGY elements in a subgroup of *Pyricularia grisea*: a possible example of successful capture of a genetic invader by a fungal genome. *Mol. Gen. Genet.* 261 958–966. 10.1007/s004380051044 10485287

[B63] Nara F. Watanabe I. Serizawa N. (1993). Development of a transformation system for the filamentous, ML-236B (compactin) producing fungus *Penicillium citrinum*. *Curr. Genet.* 23 28–32. 10.1007/BF00336746 8381335

[B64] Nielsen J. C. Grijseels S. Prigent S. Ji B. Dainat J. Nielsen K. F. (2017). Global analysis of biosynthetic gene clusters reveals vast potential of secondary metabolite production in *Penicillium* species. *Nat. Microbiol.* 2:17044. 10.1038/nmicrobiol.2017.44 28368369

[B65] Nielsen M. L. Hermansen T. D. Aleksenko A. (2001). A family of DNA repeats in *Aspergillus nidulans* has assimilated degenerated retrotransposons. *Mol. Genet. Genomics* 265 883–887. 10.1007/s004380100484 11523805

[B66] Nierman W. C. Fedorova-Abrams N. D. Andrianopoulos A. (2015). Genome sequence of the AIDS-Associated pathogen *Penicillium marneffei* (ATCC18224) and its near taxonomic relative *Talaromyces stipitatus* (ATCC10500). *Genome Announc.* 3:e01559–14. 10.1128/genomeA.01559-14 25676766 PMC4333666

[B67] Nierman W. C. Pain A. Anderson M. J. Wortman J. R. Kim H. S. Arroyo J. (2005). Genomic sequence of the pathogenic and allergenic filamentous fungus *Aspergillus fumigatus*. *Nature* 438 1151–1156. 10.1038/nature04332 16372009

[B68] Oakley C. E. Edgerton-Morgan H. Oakley B. R. (2012). Tools for manipulation of secondary metabolism pathways: rapid promoter replacements and gene deletions in *Aspergillus nidulans*. *Methods Mol. Biol.* 944 143–161. 10.1007/978-1-62703-122-6_10 23065614

[B69] Oka T. Ekino K. Fukuda K. Nomura Y. (2014). Draft genome sequence of the formaldehyde-resistant fungus *Byssochlamys spectabilis* no. 5 (anamorph *Paecilomyces variotii* no. 5) (NBRC109023). *Genome Announc.* 2:e01162–13. 10.1128/genomeA.01162-13 24407650 PMC3886963

[B70] Palmieri F. Rieder B. Ventrella A. Blanco E. Do P. T. Nunes-Nesi A. (2009). Molecular identification and functional characterization of *Arabidopsis thaliana* mitochondrial and chloroplastic NAD^+^ carrier proteins. *J. Biol. Chem.* 284 31249–31259. 10.1074/jbc.M109.041830 19745225 PMC2781523

[B71] Parra G. Bradnam K. Korf I. (2007). CEGMA: a pipeline to accurately annotate core genes in eukaryotic genomes. *Bioinformatics* 23 1061–1067. 10.1093/bioinformatics/btm071 17332020

[B72] Patil N. P. Patil K. P. Chaudhari B. L. Chincholkar S. B. (2012). Production, purification of exo-polygalacturonase from soil isolate *Paecilomyces variotii* NFCCI 1769 and its application. *Indian J. Microbiol.* 52 240–246. 10.1007/s12088-011-0162-x 23729888 PMC3386462

[B73] Peng M. Dilokpimol A. Mäkelä M. R. Hildén K. Bervoets S. Riley R. (2017). The draft genome sequence of the ascomycete fungus *Penicillium subrubescens* reveals a highly enriched content of plant biomass related CAZymes compared to related fungi. *J. Biotechnol.* 246 1–3. 10.1016/j.jbiotec.2017.02.012 28216099

[B74] Pitkin J. W. Panaccione D. G. Walton J. D. (1996). A putative cyclic peptide efflux pump encoded by the *TOXA* gene of the plant-pathogenic fungus *Cochliobolus carbonum*. *Microbiology* 142 1557–1565. 10.1099/13500872-142-6-1557 8704997

[B75] Polat M. Kara S. S. Tapisiz A. Demirtas Z. Sari S. Kalkanci A. (2015). Successful treatment of *Paecilomyces variotii* peritonitis in a liver transplant patient. *Mycopathologia* 179 317–320. 10.1007/s11046-014-9854-1 25534477

[B76] Ropars J. Dupont J. Fontanillas E. Rodríguez De La Vega R. C. Malagnac F. Coton M. (2012). Sex in cheese: evidence for sexuality in the fungus *Penicillium roqueforti*. *PLoS One* 7:e49665. 10.1371/journal.pone.0049665 23185400 PMC3504111

[B77] Samson R. A. Houbraken J. Varga J. Frisvad J. C. (2009). Polyphasic taxonomy of the heat resistant ascomycete genus *Byssochlamys* and its *Paecilomyces* anamorphs. *Persoonia* 22 14–27. 10.3767/003158509X418925 20198134 PMC2789542

[B78] Selker E. U. Garrett P. W. (1988). DNA-sequence duplications trigger gene inactivation in *Neurospora crassa*. *Proc. Natl. Acad. Sci. U.S.A.* 85 6870–6874. 10.1073/pnas.85.18.6870 2842795 PMC282080

[B79] Sherlock G. Wortman J. Chibucos M. Inglis D. Arnaud M. B. Inglis D. O. (2012). The *Aspergillus* genome database (AspGD): recent developments in comprehensive multispecies curation, comparative genomics and community resources. *Nucleic Acids Res.* 40 D653–D659. 10.1093/nar/gkr875 22080559 PMC3245136

[B80] Stamatakis A. (2014). RAxML version 8: a tool for phylogenetic analysis and post-analysis of large phylogenies. *Bioinformatics* 30 1312–1313. 10.1093/bioinformatics/btu033 24451623 PMC3998144

[B81] Steiner B. Aquino V. R. Paz A. A. da Rocha Silla L. M. Zavascki A. Goldani L. Z. (2013). *Paecilomyces variotii* as an emergent pathogenic agent of pneumonia. *Case Rep. Infect. Dis.* 2013:273848. 10.1155/2013/273848 23819077 PMC3683431

[B82] Suelmann R. Fischer R. (2000). Mitochondrial movement and morphology depend on an intact actin cytoskeleton in *Aspergillus nidulans*. *Cell Motil. Cytoskeleton* 45 42–50. 10.1002/(SICI)1097-0169(200001)45:1<42::AID-CM4>3.0.CO;2-C 10618165

[B83] Sutter R. P. (1975). Mutations affecting sexual development in *Phycomyces blakesleeanus*. *Proc. Natl. Acad. Sci. U.S.A.* 72 127–130. 10.1073/pnas.72.1.127 1054488 PMC432254

[B84] Swami T. Pannu S. Kumar M. Gupta G. (2016). Chronic invasive fungal rhinosinusitis by *Paecilomyces variotii*: a rare case report. *Indian J. Med. Microbiol.* 34 103–106. 10.4103/0255-0857.174126 26776131

[B85] Testa A. C. Oliver R. P. Hane J. K. (2016). OcculterCut: a comprehensive survey of AT-rich regions in fungal genomes. *Genome Biol. Evol.* 8 2044–2064. 10.1093/gbe/evw121 27289099 PMC4943192

[B86] Torres R. Gonzalez M. Sanhueza M. Segovia E. Alvo M. Passalacqua W. (2014). Outbreak of *Paecilomyces variotii* peritonitis in peritoneal dialysis patients after the 2010 Chilean earthquake. *Perit. Dial. Int.* 34 322–325. 10.3747/pdi.2013.00157 24584599 PMC4033333

[B87] Urquhart A. S. Idnurm A. (2017). Sit4-associated protein is required for pathogenicity of *Leptosphaeria maculans* on *Brassica napus*. *Curr. Microbiol.* 74 1438–1446. 10.1007/s00284-017-1338-3 28840344

[B88] Uzunoglu E. Sahin A. M. (2017). *Paecilomyces variotii* peritonitis in a patient on continuous ambulatory peritoneal dialysis. *J. Mycol. Med.* 27 277–280. 10.1016/j.mycmed.2017.02.001 28363816

[B89] Van de Wouw A. P. Elliott C. E. Popa K. M. Idnurm A. (2019). Analysis of Repeat Induced Point (RIP) mutations in *Leptosphaeria maculans* indicates variability in the RIP process between fungal species. *Genetics* 10.1534/genetics.118.301712 [Epub ahead of print]. 30389803 PMC6325690

[B90] van den Berg M. A. Albang R. Albermann K. Badger J. H. Daran J.-M. Driessen A. J. M. (2008). Genome sequencing and analysis of the filamentous fungus *Penicillium chrysogenum*. *Nature Biotechnol.* 26 1161–1168. 10.1038/nbt.1498 18820685

[B91] Vesth T. C. Nybo J. L. Theobald S. Frisvad J. C. Larsen T. O. Nielsen K. F. (2018). Investigation of inter- and intraspecies variation through genome sequencing of *Aspergillus* section *Nigri*. *Nat. Genet.* 50 1688–1695. 10.1038/s41588-018-0246-1 30349117

[B92] Waterhouse R. M. Seppey M. Simão F. A. Manni M. Ioannidis P. Klioutchnikov G. (2018). BUSCO applications from quality assessments to gene prediction and phylogenomics. *Mol. Biol. Evol.* 35 543–548. 10.1093/molbev/msx319 29220515 PMC5850278

[B93] Weyda I. Yang L. Vang J. Ahring B. K. Lübeck M. Lübeck P. S. (2017). A comparison of *Agrobacterium*-mediated transformation and protoplast-mediated transformation with CRISPR-Cas9 and bipartite gene targeting substrates, as effective gene targeting tools for *Aspergillus carbonarius*. *J. Microbiol. Methods* 135 26–34. 10.1016/j.mimet.2017.01.015 28159628

[B94] Zerbino D. R. Birney E. (2008). Velvet: algorithms for de novo short read assembly using de Bruijn graphs. *Genome Res.* 18 821–829. 10.1101/gr.074492.107 18349386 PMC2336801

