# Supplementary material for: Genomic and Genetic Insights Into a Cosmopolitan Fungus, Paecilomyces variotii (Eurotiales)
Source: Front Microbiol. 2018 Dec 13;9:3058. doi: 10.3389/fmicb.2018.03058 (PMC6300479; doi:10.3389/fmicb.2018.03058)
Supplement: Supplementary file 2 [file Data_Sheet_1.PDF]

FIGURE S1 | Annotations of the mitochondrial genomes of *P. variotii* strains CBS 101075 and CBS 144490. The core mitochondrial protein coding genes are colored green, RNA in yellow, tRNAs in blue, non-standard mitochondrial proteins in brown, and introns in orange. All genes are encoded on the same strand of the genome. The difference in size between the two genomes is largely a consequence of introns within *COX1* (four extra in CBS 101075) and *COB* (one extra in CBS 101075).

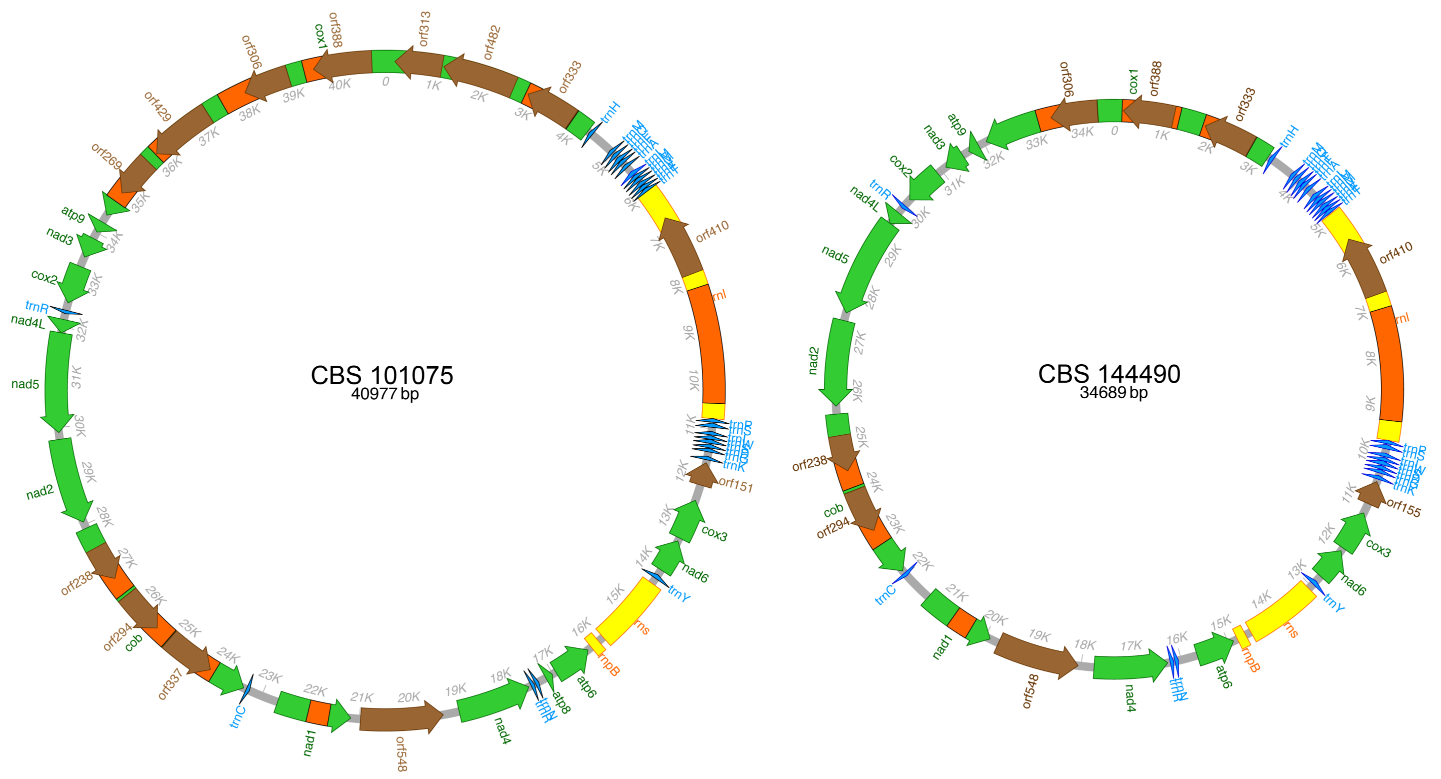

FIGURE S2 | Diagram of how the barcoding-NGS method can locate T-DNA inserts in fungal genomes. (A) DNA sequence showing the T-DNA region. The boxed TCA indicates the three nucleotides of the right border that are usually inserted into the genome, and the barcode sequence in red where N is any nucleotide and M is either A or C. (B) Overview of the position of the T-DNA (orange) within the DNA of *P. variotii* (blue). (C) Reads containing the barcode region are extracted from the sequencing data. The highlighted reads extend into the sequence flanking the T-DNA and can thus be used to determine its location in the genome via BLAST searches. The barcode allows a pool of transformed strains to be sequenced simultaneously.

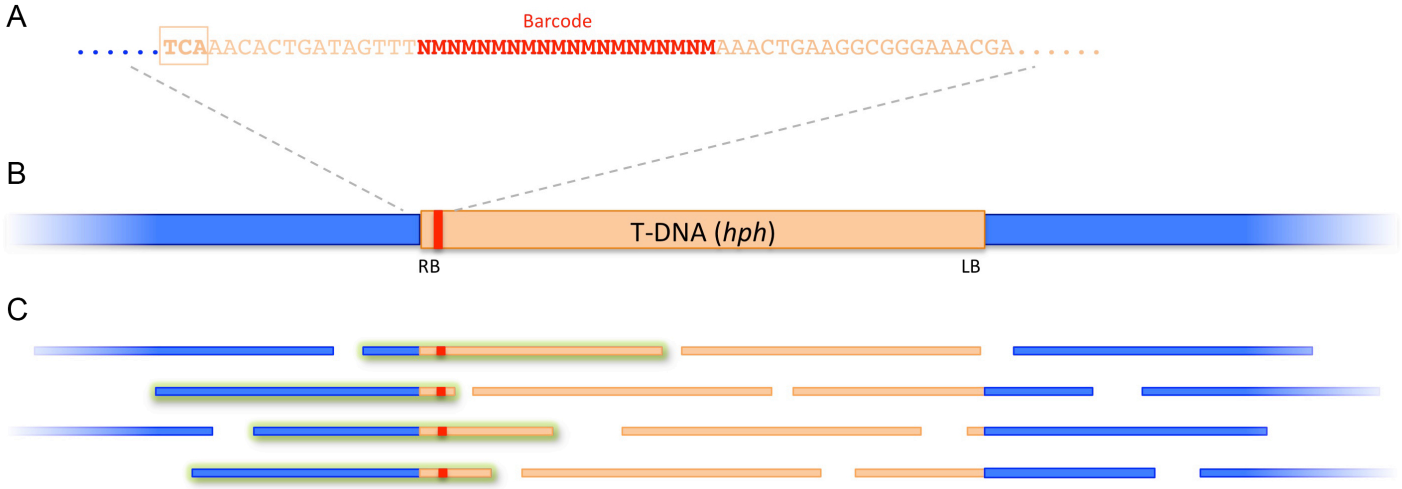

FIGURE S3 | Phylogenetic trees of strains of *P. variotii* and the related species *P. formosus* generated in MrBayes, with *P. divaricatus* and *P. nivea* as outgroups. The three regions used were (A) calmodulin, (B) β-tubulin and (C) ITS. The strain *P. variotii* no. 5 is a strain of *P. formosus*.

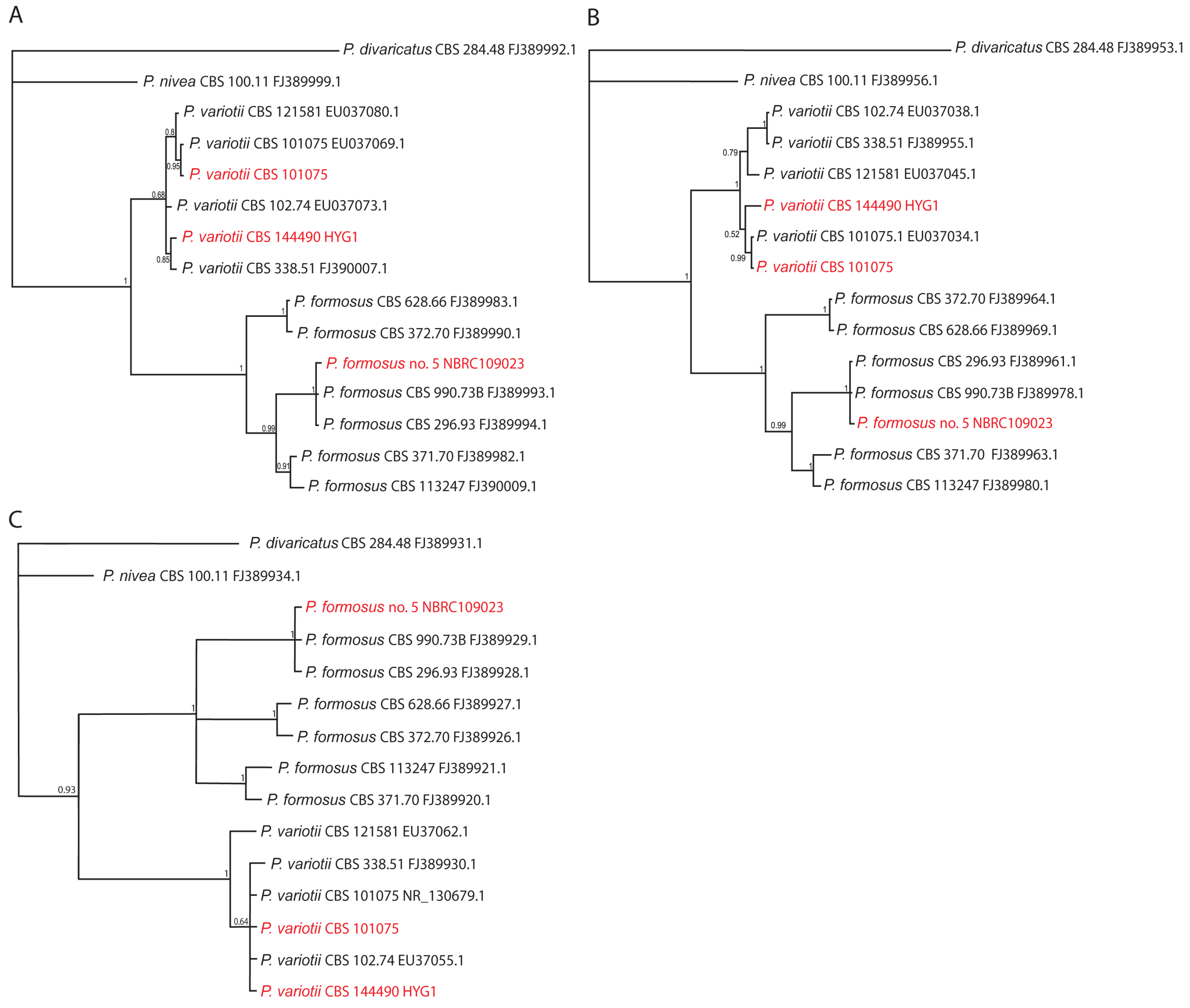

FIGURE S4 | Southern blot analysis to determine how many T-DNA insertions are present in strains AU1_63 and AU2_33. Genomic DNA of the two strains was cut with HindIII restriction enzyme, resolved on an agarose gel and blotted. The blot was probed with a DIG-labeled fragment of the *hph* open reading frame.

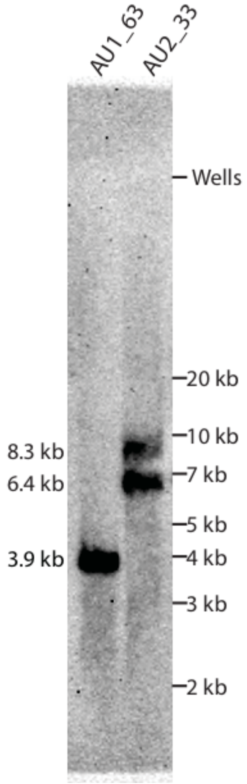

FIGURE S5 | Structure of the *MAT* locus of *P. variotii*. The unique DNA sequence and 2 kb of DNA on either side, which contains the *APN1* and *SLA2* homologs that often flank this locus in ascomycete fungi, is illustrated. Each mating type contains an idiomorphic region (grey box; 6,022 bp in CBS 101075 and 5,663 bp in CBS 144490), flanked by DNA sequences of high similarity. Tick marks indicate 500 bp intervals.

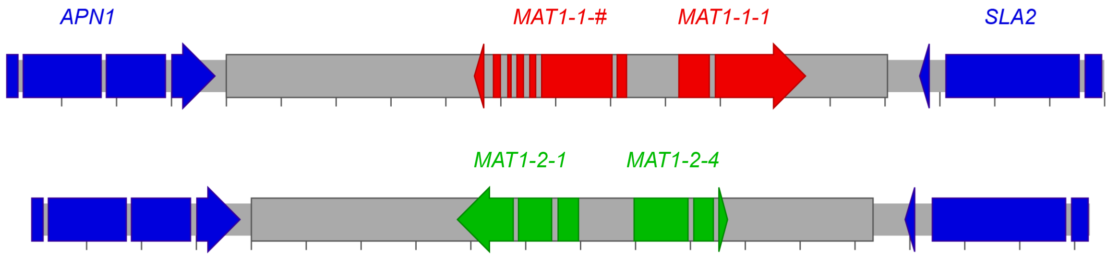

FIGURE S6 | Mutation of the *prmJ* gene does not impair sexual reproduction of *P. variotii*. (A) Production of cleistothecia (white structures) and (B) viable progeny derived from ascospores from such cleistothecia in a *prmJ*$^{-}$ × *prmJ*$^{-}$ cross. The scale bar in (A) is 1 mm, and the petri dish in (B) 10 cm in diameter.

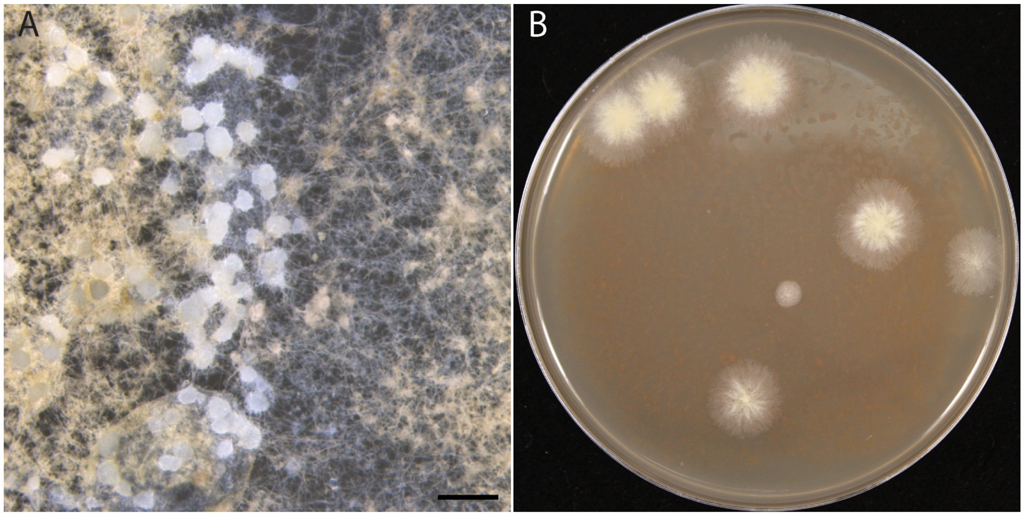

TABLE S1 | Primers used in this study.

Name	Sequence (5´-3´)
AU516	TCGAAACCTAATCAATCAACATGTCTCAAGAGCGCGAGG
AU473	CCTCCTCGCCCTTGCTCACTAGAACAGCATCCCCATCTCC
AU474	GTGAGCAAGGGCGAGGAGG
AU517	GCTCATAGTCACATCCCTCACTTGTACAGCTCGTCCATGC
AU257	CTGTCAAACACTGATAGTTTNMNMNMNMNMNMNMNMNMNMAAACTGAAGGCGG GAAACGA
AU258	CTGTCAAACACTGATAGTTT
AU259	TCGTTTCCCGCCTTCAGTTT
AU268	CTAATCGAAACCTAATCAATCAACATGGCTTCCGCTCTCCGTCTC
AU269	CACTGCGGCAGCAGCAGCGGCGCCACGGGCACCGCCGTAGACC
AU108	GCCGCTGCTGCTGCCGCAGTGAGCAAGGGCGAGGAGCT
AU68	GCTCATAGTCACATCCCTCA
AU492	TCGAAACCTAATCAATCAACATGCCTCCCAAAGCCGCTGAG
AU493	CAGCTCCTCGCCCTTGCTCACTTTGGCAGACGACGAGTACTTCG
AU494	GTGAGCAAGGGCGAGGAGCTG
AU495	GCTCATAGTCACATCCCTC
AU436	TCTCGAGAGGACGGATATCC
AU438	CGTTTTAACCCTGGTCTCTG
AU437	CAGAGACCAGGGTTAAAACG
AU440	CAACCCATGCCCCTGTGTGC
AU462	AGCTCGGTACCCGGGGATCCTCTAGACAACCCATGCCCCTGTGTGC
Match2R	TTTCTGCGATATGATTATCCG
AU448	GCCCAGGTTGGCAGGTAACC
MAI0440	CTGGGATTGCCCCTCGATGC
MAI0441	CCTACTGAACGTTATGAC
MAI0442	AACAGCTATGACATGATTACGGGATTTCGGATCTACAGC
MAI0443	GCATCGAGGGGCAATCCCAGAGATGGAGAGGCTGGAAC
MAI0444	GTCATAACGTTCAGTAGGAGGCTAGTTTGATTGCTC
MAI0445	GTAAAACGACGGCCAGTGCCATTTATTGAAGACCGTGGG
AU268	CTAATCGAAACCTAATCAATCAACATGGCTTCCGCTCTCCGTCTC
AU68	GCTCATAGTCACATCCCTCA
AU439	TGATCTCGTCTAGCCGTTGG
FD1212AF	ATGGCTGGCGGCTATTTTG
FD1212ER	GATCCGGCAACAATACTGC
FD1212DF	GCAGTATTGTTGCCGGATC
FD1212FR	CTAAAAGCTGAAAAGTCCAC
FD1212AFPLAU2	TCGAAACCTAATCAATCAACATGGCTGGCGGCTATTTTG
FD1212FRPLAU2	TGCTCATAGTCACATCCCTCAAAAGCTGAAAAGTCCAC
AU446	ACACGCACATTCACTCTACG
AU448	GCCCAGGTTGGCAGGTAACC
AU461	CTAGGCCTCTGCAGGTCGACTCTAGATCTCGAGAGGACGGATATCC
MitoMarkerF	AAGTGTCAATATAAAATACC
MitoMarkerR	TTATTAGCAATGGTTGGAGC
Match2Fconserved	GGTCTATCGTGAGCTCGTCC
Match2R	TTTCTGCGATATGATTATCCG
123NTSF	TTCTGTATCTGCGGATCTGC
123NTSR	GAATAGGTCTATCGAAGACC
123NTEF	GGATTACCCGGGTGTAATGG
123NTER	CTCCTATAAATACTATTCGC
ai076	AACAGTTGCGCAGCCTGAATG

TABLE S2 | Sequences of barcodes generated.

Number	Barcode sequence
CSB1	TCTTGCTTGATCTCTCTCTC
CSB2	TCTATATTGCGAGTGATCTG
CSB4	GTGTGCTCGCGCGGTGGTGC
CSB10	TATGTATTTTTAGTGTTGTT
CSB11	GGTCTGTATCTATAGATTTG
CSB13	GAGATTTAGGGTGATATAGG
CSB14	GTTATTTTTTGTTGTGGAGC
CSB15	TCTATTGAGATTGATATAGA
CSB16	TTGGGATCTTTATGGGGGGG
CSB18	TATGTGGATAGAGTGTGAGC
CSB20	GATCTATATATCTATTGAGC

TABLE S3 | Genetic markers used for segregation analysis in *P. variotii* crosses.

Marker	Forward primer	Reverse primer	Restriction enzyme	Expected DNA fragment sizes (bp)	
				CBS 144490	CBS 101075
Mitochondrial Marker	Mitomarker F	Mitomarker R	NcoI	120, 510, 237	630; 237
Mating type	MAI0448 MAI0449	MAI0450 MAI0451	none	231	340
123A	123NTSF	123NTSR	BamHI	332, 278	613
123B	123NTEF	123NTER	BamHI	447	210, 237

TABLE S4 | Genome assembly and gene content statistics for the two *P. variotii* strains compared to four other Eurotiales species.

	***P. variotii* CBS 101075**		***P. variotii* CBS144490 HYG1**		***B. spectabilis***		***A. steynii***		***P. solitum***		***T. lanuginosus***	
**# contigs**	86		126		1,053		37		597		311	
**Assembly length**	30,105,809		32,365,222		29,762,401		37,847,960		33,184,895		19,155,516	
**Contig N50**	5		22		72		4		32		53	
**Contig L50**	1,732,371		447,384		137,200		3,921,250		320,498		113,553	
**Percent repeats**	4.53%		5.73%		1.89%		5.96%		4.65%		1.00%	
**Percent CEGMA**	100%		99.78%		99.34		99.34		98.03		97.82	
**BUSCO (Eurotiomycetes)**												
**Single copy**	96.4%		96.2%		96.4%		95.9%		97.0%		87.2%	
**Duplicated**	0.2%		0.2%		0.2%		0.2%		0.4%		0.2%	
**Fragmented**	1.8%		1.7%		1.5%		2.1%		1.3%		4.1%	
**Total**	98.4%		98.1%		98.1%		98.2%		98.7%		91.5%	
**BUSCO (Fungi)**												
**Single copy**	98.6%		97.9%		97.6%		97.6%		97.2%		94.1%	
**Duplicated**	0.7%		1.0%		0.0%		0.7%		0.7%		0.3%	
**Fragmented**	0.3%		0.3%		1.4%		0.0%		0.0%		1.0%	
**Total**	99.6%		99.2%		99.0%		98.3%		97.9%		95.4%	
**BUSCO (Eukaryota)**												
**Single copy**	98.3%		98.3%		97.4%		96.7%		99.0%		94.4%	
**Duplicated**	0.7%		0.7%		0.3%		1.0%		0.3%		0.0%	
**Fragmented**	0.0%		0.0%		1.7%		1.0%		0.0%		0.7%	
**Total**	99.0%		99.0%		99.4%		98.7%		99.3%		95.1%	
**# genes**	9,270	100%	9,230	100%	8,877	100%	9,886	100%	11,396	100%	6,241	100%
**Hits to databases:**												
**NCBI nr**	8,825	95.20%	8,853	95.92%	8,469	95.40%	9,150	92.56%	11,101	97.41%	6,028	96.59%
**HMMPfam**	6,954	75.02%	6,977	75.59%	7,034	79.24%	6,176	62.47%	6,949	60.98%	5,133	82.25%
**HMMPfam unique**	3,496	38%	3,491	38%	3,485	39%	2,463	25%	3,564	31%	3,272	52%
**# cds complete genes**	9,155	98.76%	8,931	96.76%	8,789	99.01%	9,617	97.28%	11,161	97.94%	6,160	98.70%
**Proteins with transmembrane helices**	1,937	20.90%	1,883	20.40%	1,853	20.87%	2,119	21.43%	2,171	19.05%	1,160	18.59%
**Proteins with signal peptides**	1,753	18.91%	1,728	18.72%	1,609	18.13%	2,083	21.07%	2058	18.06%	988	15.83%

Genomes for comparison:
Byssochlamys spectabilis No. 5 (Oka et al., 2014)
Aspergillus steynii IBT 23096 (Kjaerbolling et al., 2018)
Penicillium solitum IBT 29525 (Nielsen et al., 2017)
Thermomyces lanuginosus SSBP (McHunu et al., 2013)

TABLE S5 | Location of T-DNA inserts determined from analysis of next generation sequencing of the pool of all seven *P. variotii* strains.

<table>
<tr><th>Strain</th><th>Barcode</th><th>Strain background</th><th>Locations of T-DNA insertions in genome</th></tr>
<tr><td>JSCS3</td><td>none</td><td>CBS 101075</td><td>Scaffold 6, Scaffold 8</td></tr>
<tr><td>AU2_33</td><td>2</td><td>CBS 144490</td><td>Scaffold 2, Scaffold 2</td></tr>
<tr><td>AU10_6</td><td>10</td><td>CBS 144490</td><td>Scaffold 1, Scaffold 4</td></tr>
<tr><td>AU4_W</td><td>4</td><td>CBS 101075</td><td>None found</td></tr>
<tr><td>AU1_63</td><td>1</td><td>CBS 101075</td><td rowspan="3">Scaffold 2, Scaffold 1</td></tr>
<tr><td>AU_S1</td><td>1</td><td>CBS 101075</td></tr>
<tr><td>AU_C1</td><td>1</td><td>CBS 101075</td></tr>
</table>

TABLE S6 | Segregation of traits in progeny from an AU1_63 × CBS 144490 cross. "C" indicates that the PCR product was cleaved by the restriction enzyme and "UC" indicates that it was not.

**Strain**	**123A**	**123B**	**Mitochondrial marker**	**Mating type**	**Colony phenotype**	**Hygromycin sensitivity**
CBS 101075	UC	C	3	MAT1-1	wild type	Sensitive
CBS 144490	C	UC	2	MAT1-2	wild type	Sensitive
AU1_63	UC	C	3	MAT1-1	Pale	Resistant
1	UC	UC	2	MAT1-2	wild type	Sensitive
2	C	UC	2	MAT1-2	wild type	Sensitive
3	C	C	3	MAT1-1	wild type	Sensitive
4	UC	C	2	MAT1-2	wild type	Sensitive
5	C	UC	2	MAT1-2	wild type	Sensitive
6	C	UC	2	MAT1-2	wild type	Sensitive
7	C	UC	3	MAT1-2	wild type	Sensitive
8	UC	UC	3	MAT1-1	wild type	Sensitive
9	UC	C	3	MAT1-1	wild type	Sensitive
10	UC	C	3	MAT1-2	wild type	Sensitive
11	C	C	3	MAT1-1	wild type	Sensitive
13	C	C	3	MAT1-2	wild type	Sensitive
14	C	UC	3	MAT1-1	wild type	Sensitive
15	UC	UC	3	MAT1-1	wild type	Sensitive
16	C	UC	3	MAT1-2	Pale	Resistant
17	C	C	3	MAT1-2	Pale	Resistant
18	UC	C	3	MAT1-1	Pale	Resistant
19	UC	C	3	MAT1-1	Pale	Resistant
20	C	UC	3	MAT1-1	Pale	Resistant
21	C	UC	3	MAT1-2	Pale	Resistant
22	UC	UC	3	MAT1-2	Pale	Resistant
23	UC	UC	3	MAT1-1	Pale	Resistant
24	C	C	3	MAT1-1	Pale	Resistant
25	UC	UC	3	MAT1-2	Pale	Resistant
26	UC	C	3	MAT1-1	Pale	Resistant
27	C	C	3	MAT1-2	Pale	Resistant
28	UC	UC	3	MAT1-2	Pale	Resistant
29	UC	C	3	MAT1-1	Pale	Resistant
30	C	UC	3	MAT1-1	Pale	Resistant
31	UC	C	3	MAT1-2	Pale	Resistant

TABLE S7 | Co-segregation of *dspA* alleles with phenotypes in a AU2_33 × CBS 101075 cross.

**Strain**	***dspA* gene** (+) Present (-) Mutant	**Second T-DNA** (+) Absent (-) Present	**Mitochondrial marker**	**Mating type**	**Colony phenotype**	**Hygromycin sensitivity**
CBS 144490	+	+	2	MAT1-2	Normal	Sensitive
CBS 101075	+	+	3	MAT1-1	Normal	Sensitive
AU2_33	-	-	2	MAT1-2	Delayed	Resistant
2	-	-	3	MAT1-2	Delayed	Sensitive
3	-	-	3	MAT1-2	Delayed	Sensitive
4	-	-	3	MAT1-1	Delayed	Sensitive
5	-	+	3	MAT1-1	Delayed	Sensitive
6	-	-	3	MAT1-2	Delayed	Sensitive
8	-	-	3	MAT1-2	Delayed	Sensitive
9	-	-	3	MAT1-2	Delayed	Sensitive
10	-	-	3	MAT1-2	Delayed	Sensitive
7	+	+	3	MAT1-1	Normal	Sensitive
11	+	+	3	MAT1-1	Normal	Sensitive
12	+	+	3	MAT1-2	Normal	Sensitive
13	+	+	3	MAT1-2	Normal	Sensitive
14	+	+	3	MAT1-2	Normal	Sensitive
15	+	+	3	MAT1-2	Normal	Sensitive
16	+	+	3	MAT1-1	Normal	Sensitive
17	-	-	3	MAT1-2	Delayed	Sensitive
18	+	+	3	MAT1-1	Normal	Sensitive
19	-	-	3	MAT1-2	Delayed	Sensitive
20	+	+	3	MAT1-1	Normal	Sensitive
1	+	+	3	MAT1-2	Normal	Sensitive

TABLE S8 | Comparison of gene numbers in the top 100 clusters for *P. variotii* and other ascomycete species. Each row is colored from highest (yellow) to lowest (blue) number of genes for each species.

JGI cluster number	*Aspergillus aculeatinus*	*Aspergillus bombycis*	*Aspergillus calidoustus*	*Aspergillus fijiensis*	*Aspergillus homomorphus*	*Aspergillus ibericus*	*Aspergillus nidulans*	*Aspergillus uvarum*	*Byssochlamys spectabilis* No. 5	*Nectria haematococca*	*Neurospora crassa*	*Paecilomyces variotii* CBS 101075	*Paecilomyces variotii* CBS 144490	*Penicillium griseofulvum*	*Penicillium steckii*	*Penicillium subrubescens*	*Thermoascus aurantiacus*
**1**	60	84	133	59	59	64	79	61	51	115	28	46	44	65	104	117	44
**2**	49	79	84	53	50	56	57	47	59	96	33	44	45	42	65	64	35
**3**	62	71	73	59	68	70	55	58	31	100	41	34	33	42	42	47	28
**4**	69	82	58	66	69	63	45	71	42	48	16	31	33	49	52	71	21
**5**	63	65	74	64	55	61	56	54	42	79	16	32	35	48	49	55	30
**6**	33	66	92	33	29	31	52	31	31	92	20	25	24	34	48	52	34
**7**	41	53	49	41	37	31	33	38	25	46	17	18	18	40	37	38	19
**8**	25	31	40	26	24	24	25	26	25	58	15	17	18	30	33	26	16
**9**	31	41	22	31	31	35	24	29	16	38	6	14	14	29	41	37	15
**10**	30	41	32	31	32	33	22	29	19	42	8	16	15	22	27	38	11
**11**	12	19	22	11	11	13	13	11	23	87	30	24	30	11	22	17	41
**12**	23	28	29	22	21	22	27	25	16	46	10	9	12	21	24	28	13
**13**	25	30	38	25	19	19	22	25	14	42	2	10	10	23	25	27	12
**14**	32	22	34	32	25	25	15	33	6	49	5	7	6	14	17	21	8
**15**	9	30	82	12	12	8	9	12	1	29	8	3	3	22	31	72	7
**16**	12	22	58	12	9	11	21	15	8	68	6	4	4	16	24	52	4
**17**	18	18	27	18	18	19	19	18	17	24	25	19	19	16	19	23	20
**18**	21	29	30	20	20	25	22	19	19	19	3	16	15	21	18	24	11
**19**	22	25	24	20	13	15	15	19	19	35	7	15	11	16	21	23	13
**20**	19	25	28	17	36	38	6	20	8	16	6	23	23	13	7	10	10
**21**	19	27	36	21	14	16	23	15	17	35	5	14	11	16	15	11	9
**22**	11	23	65	15	15	19	26	16	4	22	4	2	8	7	22	33	5
**23**	11	21	29	12	9	17	16	16	15	35	5	12	13	10	20	34	8
**24**	25	20	22	22	19	16	15	21	14	23	5	12	10	13	14	21	7
**25**	23	22	27	27	13	21	13	22	12	26	3	6	5	16	13	24	5
**26**	21	21	28	24	29	20	18	24	3	11	5	7	7	17	8	24	5
**27**	25	23	12	28	17	23	12	25	10	17	9	9	9	15	8	17	8
**28**	19	23	11	21	16	15	9	20	7	34	2	5	5	17	25	21	3
**29**	15	18	22	15	13	16	17	17	12	23	7	12	12	12	13	17	11
**30**	15	18	28	12	7	15	12	13	14	42	6	11	11	12	9	13	10
**31**	18	11	31	19	25	27	5	24	10	3	0	20	18	13	5	4	8
**32**	15	16	34	14	10	15	11	13	5	28	9	7	7	9	12	23	7
**33**	16	17	26	16	12	15	14	14	6	38	5	6	7	6	11	11	5
**34**	13	14	14	13	11	15	14	12	11	14	15	12	12	13	13	15	11
**35**	14	10	13	15	14	12	13	15	12	14	13	13	13	11	10	13	12
**36**	15	20	22	15	14	12	8	14	12	24	6	7	7	11	8	13	7
**37**	21	16	18	21	11	16	12	22	6	6	4	7	6	13	14	13	7
**38**	15	14	18	15	12	10	10	13	12	23	4	11	11	7	7	14	15
**39**	22	16	21	25	20	26	9	23	5	17	1	2	2	2	8	7	2
**40**	20	18	16	18	12	8	5	20	10	24	5	6	5	7	8	17	5
**41**	11	11	13	12	14	13	13	12	8	14	13	10	10	13	11	14	10
**42**	16	18	13	16	9	13	12	13	7	10	4	9	8	16	7	18	12
**43**	15	14	13	15	10	20	9	15	11	22	6	6	7	9	11	9	6
**44**	12	16	20	11	12	10	16	12	10	13	6	5	6	9	11	10	12
**45**	10	14	24	8	8	12	12	6	12	17	2	9	9	8	14	17	9
**46**	15	17	21	15	17	16	5	16	6	10	1	9	11	7	6	10	5
**47**	13	20	16	12	12	9	10	10	7	22	2	6	6	9	18	7	6
**48**	9	12	12	10	9	11	11	11	10	24	4	8	8	12	11	13	6
**49**	13	12	26	12	10	14	16	8	7	15	3	7	7	6	9	5	4
**50**	9	18	12	9	10	13	14	8	13	12	4	6	6	7	13	12	7
**51**	15	15	15	15	11	11	12	13	4	14	4	2	3	14	11	8	2
**52**	0	8	1	0	0	6	2	2	0	108	31	0	0	0	10	1	0
**53**	13	13	11	13	10	11	12	10	6	10	6	8	9	7	7	9	5
**54**	16	12	11	17	11	17	8	16	3	18	1	4	4	4	6	7	5
**55**	10	19	12	12	10	8	8	8	5	16	8	4	4	9	9	12	4
**56**	12	11	10	14	11	8	8	11	9	6	0	8	8	8	18	12	4
**57**	8	4	24	12	13	9	11	11	2	19	0	1	1	5	17	17	3
**58**	10	9	7	10	9	12	5	10	6	15	9	7	7	7	8	17	6
**59**	8	10	14	8	4	5	10	8	11	27	4	6	6	5	11	10	3
**60**	9	12	10	10	8	8	10	7	7	8	5	7	7	9	10	12	9
**61**	15	11	13	13	13	11	7	11	8	6	1	6	7	6	8	8	4
**62**	7	14	9	7	7	10	5	7	10	21	5	8	8	9	7	12	1
**63**	8	11	10	7	8	10	9	7	11	21	2	8	8	7	8	8	3
**64**	14	17	4	19	16	5	9	14	3	3	1	1	1	10	6	21	1
**65**	6	11	16	6	6	11	10	6	9	13	4	8	8	7	7	12	4
**66**	4	12	26	4	4	5	6	4	5	34	2	3	3	7	9	13	3
**67**	0	18	0	0	0	10	2	0	0	95	15	0	1	0	1	0	0
**68**	8	15	18	9	9	12	6	8	7	8	2	6	6	5	9	7	3
**69**	10	10	11	10	8	6	8	8	8	13	4	4	4	8	7	12	5
**70**	8	8	8	8	8	8	8	8	8	8	8	8	8	8	7	8	8
**71**	9	9	15	10	9	8	7	10	1	27	3	1	1	7	5	10	1
**72**	13	6	11	11	7	8	10	12	5	15	3	5	4	3	8	9	3
**73**	8	12	14	8	11	9	8	8	5	8	7	4	4	5	11	7	4
**74**	8	16	14	8	10	6	15	6	4	12	4	1	1	4	11	9	1
**75**	9	14	13	9	7	9	8	9	2	9	0	2	2	7	10	18	2
**76**	7	5	9	10	7	10	7	9	5	17	1	7	6	9	5	10	4
**77**	7	15	23	5	3	3	4	3	6	12	1	7	6	7	9	15	1
**78**	10	8	5	10	8	8	3	10	7	9	10	6	6	5	10	7	5
**79**	7	9	12	7	4	6	5	6	10	12	3	9	7	4	9	10	6
**80**	9	6	12	11	9	9	10	12	5	10	5	3	4	2	6	10	2
**81**	8	11	12	7	5	9	6	7	10	8	3	9	9	4	4	8	3
**82**	8	12	15	7	4	7	5	8	5	18	2	2	3	7	4	12	1
**83**	11	12	10	11	12	8	7	13	3	7	3	2	2	7	3	4	4
**84**	10	13	5	10	10	8	4	8	4	12	1	3	3	2	10	10	5
**85**	8	10	10	7	10	6	3	8	3	8	2	4	4	11	7	11	3
**86**	6	10	13	6	6	4	7	6	4	11	3	4	5	6	8	10	4
**87**	10	9	7	8	8	4	8	11	5	9	2	4	4	6	5	8	5
**88**	7	6	8	9	6	7	6	8	6	7	7	6	6	5	3	8	6
**89**	11	8	13	11	7	5	10	11	2	6	5	1	1	4	5	8	2
**90**	10	7	7	11	12	4	6	13	2	1	1	3	3	11	9	7	2
**91**	6	6	8	6	6	6	7	6	6	7	5	7	7	5	7	7	7
**92**	6	9	10	6	6	9	5	6	4	6	7	4	4	7	7	6	5
**93**	6	12	8	6	4	6	8	5	4	11	2	3	3	7	7	8	5
**94**	6	7	8	6	6	5	7	6	6	8	4	6	5	5	6	7	7
**95**	4	6	22	5	3	3	7	6	1	28	1	1	1	3	4	9	0
**96**	9	7	11	7	6	4	5	7	7	11	1	5	5	3	5	7	3
**97**	7	11	7	7	7	11	8	6	3	9	3	3	3	4	5	6	3
**98**	6	6	6	6	6	6	6	6	6	6	7	6	6	6	6	6	6
**99**	6	6	7	6	6	6	6	6	6	7	7	6	6	4	6	5	6
**100**	6	5	17	8	7	7	3	4	1	12	2	1	1	6	7	11	2
